# Intermittent fasting and immune aging: implications for immunosenescence, inflammaging, neuroinflammation, and frailty

**DOI:** 10.3389/fnut.2026.1736969

**Published:** 2026-01-21

**Authors:** Dania Alkawamleh, Mohamed I. Madkour, Faiza Kalam, Dana N. Abdelrahim, Hanan Wael Abdallah, MoezAlIslam E. Faris

**Affiliations:** 1 Department of Nutrition, King Hussein Cancer Center, Amman, Jordan; 2 Department of Clinical Nutrition and Dietetics, Faculty of Allied Medical Sciences, Applied Science Private University, Amman, Jordan; 3 Department of Medical Laboratory Sciences, College of Health Sciences, University of Sharjah, Sharjah, United Arab Emirates; 4 Research Institute of Medical and Health Sciences (RIMHS), University of Sharjah, Sharjah, United Arab Emirates; 5 Division of Cancer Prevention and Control, Department of Internal Medicine, The Ohio State University, Columbus, OH, United States; 6 Ohio State University Comprehensive Cancer Center, The Ohio State University, Columbus, OH, United States

**Keywords:** AMPK activation, healthy aging, immune resilience, inflammasome, mTOR signaling, time-restricted eating

## Abstract

Aging is accompanied by a progressive decline in immune function, known as immunosenescence, and by a chronic low-grade inflammatory state, termed inflammaging. Both conditions contribute to increased susceptibility to infections, reduced vaccine responses, and the development of age-related diseases. Emerging evidence suggests that intermittent fasting (IF), a dietary pattern that alternates between periods of fasting and feeding, may influence pathways associated with immune aging across mid-life and older adulthood. This review explores how IF may exert immunoregulatory effects through metabolic remodeling, cellular stress responses, and inflammatory signaling. Preclinical and human studies indicate that IF attenuates pro-inflammatory cytokine production, enhances autophagy, and improves immune cell function, potentially delaying immunosenescence and reducing inflammaging in middle-aged and older populations. Additionally, IF may protect against neuroinflammation and cognitive decline by reducing oxidative stress, activating AMPK-SIRT1 and ketone signaling via β-hydroxybutyrate (BHB), enhancing neuroplasticity, upregulating brain-derived neurotrophic factor, and suppressing pro-inflammatory cytokines, inflammation, and frailty in the aging brain. However, most evidence comes from short- to medium-term studies in selected, relatively healthy populations, with benefits often similar to those of continuous calorie restriction, and there is limited data on long-term safety, adverse effects, and outcomes in frail older adults. By reducing oxidative stress and inflammaging, IF may mitigate frailty in older adults or delay its progression when initiated earlier. By integrating insights from immunometabolism and gerontology, this review highlights the potential role of IF as a non-pharmacological strategy to promote healthy immune aging and support functional outcomes in older adults. However, evidence in frail older adults remains limited, and randomized trials in this population are warranted. Future research should directly compare IF with isocaloric non-fasting regimens, include long-term follow-up, and carefully characterize safety and adherence in high-risk groups before IF can be routinely recommended for immune aging.

## 1 Introduction

By 2030, the World Health Organization (WHO) estimates that one in six people globally will be aged 60 or older, up from 1.4 billion in 2020. By 2050, the population over the age of 60 is expected to double to 2.1 billion, and the number of individuals over the age of 80 is estimated to triple to 426 million (1). Due to the rapid aging of the global population, there is increasing interest in strategies to delay or reverse the adverse consequences of aging, including frailty and immune aging.

Aging is associated with a progressive increase in chronic disease burden, disability, and frailty, which collectively reduce quality of life and increase mortality risk (2). Aging is a complex, multifactorial biological process characterized by the progressive decline in physiological integrity, loss of structure and function of cells, tissues, and organs, increased vulnerability to disease, and diminished capacity to adapt to stress (3). The representative hallmarks of aging, such as altered intracellular communication, stem cell exhaustion, and systemic inflammation, collectively contribute to a functional decline in various organ systems, including the immune system (4). Frailty, a clinically relevant geriatric syndrome, reflects increased vulnerability of older adults to everyday stressors. This reduced physiological reserves and the age-related gradual deterioration of multiple physiological systems (5). Frailty is of particular concern because it increases the risk of premature death and is associated with adverse outcomes, such as falls, fractures, dementia, disability, impaired quality of life, and greater reliance on health resources (6).

Immune aging, the gradual decline in the immune system’s ability to fight infections and heal as people age, drives frailty through two interconnected mechanisms: immunosenescence and inflammaging. Immunosenescence, the decline of innate and adaptive immune function, reduces vaccine efficacy and increases susceptibility to infections, age-related diseases, and cancers (7). At the same time, inflammaging refers to chronic, low-grade, sterile systemic inflammation, an inflammation without infection, that occurs with physiological aging, characterized by elevated subclinical levels of inflammatory markers such as TNF-α, IL-6, IL-1β, CRP, serum amyloid A, and fibrinogen (8). Besides, inflammaging has been identified as a hallmark of aging (9).

Nutritional approaches aimed at modulating immune function, referred to as immunonutrition, use antioxidant-rich diets, omega-3 fatty acids, and key micronutrients to help alleviate chronic inflammation, neuroinflammation, enhance neuroplasticity and induce neuroprotection, and slow age-related immune decline (10). Additionally, calorie restriction (CR) is among the most extensively studied dietary interventions in aging research. It has shown robust effects in enhancing immune surveillance, reducing systemic inflammation, and preserving cognitive function (10, 11). Compared to CR, intermittent fasting (IF) has emerged as a more practical and promising non-pharmacological approach that mimics many of CR’s immunometabolic benefits. IF has been shown to modulate key aging-related pathways, including those involved in inflammation, oxidative stress, autophagy, and immune cell renewal (12). However, most mechanistic evidence comes from animal models or short- to medium-term trials in relatively healthy adults, and it remains uncertain whether IF confers durable benefits for clinically relevant outcomes, such as inflammaging or validated frailty indices, in older and frail populations (13). Through these mechanisms, IF may enhance immune resilience and reduce the burden of immunosenescence and inflammaging, potentially delaying or mitigating frailty in aging populations (12). Though at present, it should be regarded as a promising candidate strategy rather than an established intervention for frailty and immune aging. Throughout this review, mechanistic findings from animal models are explicitly identified as such and are not assumed to translate directly into clinical benefit; where human data are available, they are reported separately and critically appraised.

This review explores the impact of dietary interventions such as IF on modulating immune aging, with a specific focus on their implications for frailty in older adults. By examining mechanistic insights and current evidence, we aim to provide a comprehensive understanding of how nutritional modulation can promote immune resilience and healthy longevity.

## 2 Overview of intermittent fasting, terminology, types, and practices

Fasting is broadly defined as “a voluntary abstinence from some or all foods or foods and beverages for preventive, therapeutic, religious, cultural, or other reasons.” Fasting can be a fluids-only regimen, in which only beverages are permitted for a specified period. Fluids-only regimens generally allow water, unsweetened herbal drinks, and broths. Some variants permit limited energy intake (e.g., small amounts of fruit juice, up to ~500 kcal/day), but these are not strictly ‘zero-calorie’ fasts. Dry fasting is a form of complete fasting from all foods and beverages for a specified duration (14).

**Figure 1:**
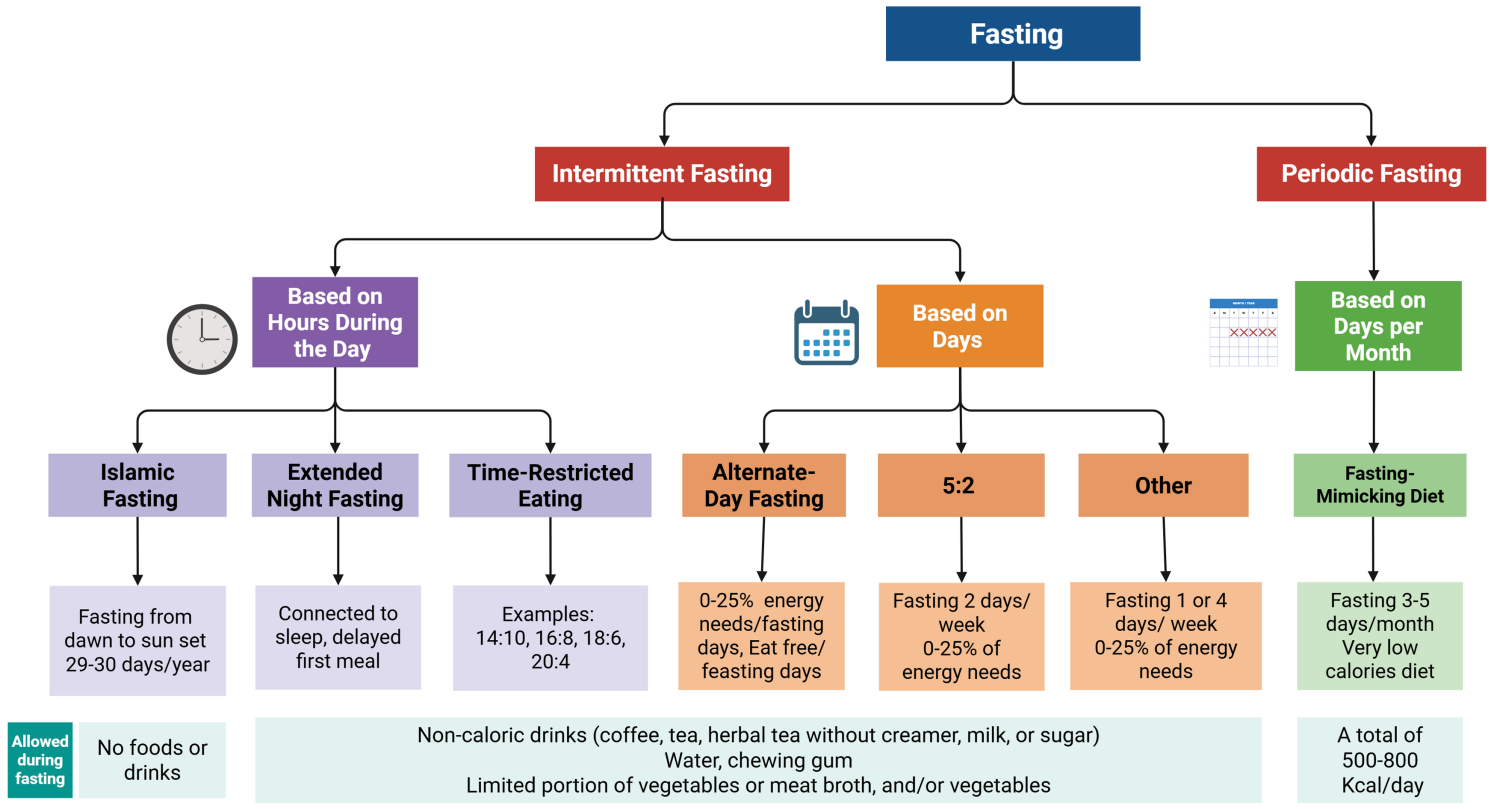
Schematic summary of the different fasting regimens.

IF refers to recurrent fasting intervals of up to ~48 h (14). There are several variants of IF, including time-restricted eating (TRE), alternate-day fasting (ADF), the 5:2 diet, and periodic fasting (PF), also known as fasting mimicking diet (FMD). TRE involves a daily eating window of 6–12 h and fasting for the rest of the day. Early TRE (eTRE) refers to earlier windows (e.g., 7 a.m.–3 p.m.), while late TRE (lTRE) refers to later windows (e.g., 12 p.m.–8 p.m.). ADF involves alternating between fasting and regular eating days. The 5:2 diet involves restricting intake to two days per week, while eating normally on the other five. PF or FMD consists of limiting food intake to a low-calorie level for five consecutive days once per month (15). Furthermore, various forms of fasting are also practiced for spiritual and cultural purposes. Ramadan intermittent fasting (RIF), or Islamic fasting, involves abstaining from food, drink, and certain behaviors from dawn to sunset during the holy month. Other religions, including Christianity, Buddhism, Jainism, Hinduism, and Judaism, incorporate fasting in various ways (16). Variations of fasting and IF regimens are illustrated in Figure 1. In clinical studies, however, these nominal fasting patterns are not always implemented in isolation. Some regimens are eucaloric, maintaining usual energy intake within the feeding window. In contrast, others combine IF with caloric restriction, such as reducing energy intake to ≤25% of requirements or implementing a hypocaloric diet on fasting days. Additionally, many protocols may modify meal composition or macronutrient distribution, such as low-carbohydrate, ketogenic, or Mediterranean diet (13, 17). As a result, the observed effects of fasting may be confounded by changes in total energy intake or nutrient profile, highlighting the complexity of interpreting outcomes. Throughout this review, individual studies are therefore described according to their specific fasting protocols to facilitate interpretation of their findings.

## 3 Immunosenescence and inflammaging: hallmarks of immune aging

### 3.1 Immunosenescence

Immunosenescence, the aging of the immune system, is closely linked to inflammaging. It is marked by both qualitative and quantitative changes in immune cell populations. Hallmarks of immunosenescence include a decrease in naïve T and B lymphocytes, resulting in diminished immune resistance to pathogens with aging and reduced vaccine efficacy (18). This decline is due to thymic involution, the gradual shrinkage and functional deterioration of the thymus, which reduces the output of naïve T cells over time (7). Aging skews the peripheral pool of T and B lymphocytes toward memory phenotypes, reducing diversity and responsiveness to novel antigens, thereby limiting effective immune surveillance and impairing the ability to generate robust responses to vaccines and emerging infections (18).

Another hallmark of immunosenescence is the accumulation of senescent immune cells, particularly CD8 + T-cells, that exhibit impaired proliferative capacity and altered cytokine profile (7). This accumulation is characterized by the development of a senescence-associated secretory phenotype (SASP), which secretes numerous soluble factors, including IL-1β, IL-6, IL-8, IL-13, IL-18, and TNF-α, as well as their receptors, resulting in a process known as inflammaging (7). Inflammaging further disrupts immune regulation, contributing to the development of age-related diseases. Together, these alterations compromise immune surveillance and responsiveness, increasing vulnerability to infections, cancer, and chronic inflammatory conditions in the elderly (19).

The aging immune system is characterized by metabolic alterations, including increased glycolysis, mitochondrial dysfunction, and increased production of reactive oxygen species (ROS) (7). These metabolic changes of immunosenescence are significantly linked to higher rates of morbidity and mortality from age-associated diseases such as cardiovascular and neurodegenerative diseases, autoimmune disorders, metabolic diseases, and cancers in older patients (7). Senescent cells exhibit irreversible cell cycle arrest, abnormal morphology, diminished capacity to divide, and resistance to the limiting effects of persistent antigens, thereby accumulating DNA damage (7).

### 3.2 Inflammaging

Inflammaging is further characterized by a complex interplay of cellular and molecular mechanisms that sustain chronic immune activation during aging (20). Multiple interconnected mechanisms contribute to inflammaging, including persistent exposure to antigenic stressors, oxidative stress, gut microbiota dysbiosis, sedentary lifestyle, a high-fat diet, and abdominal adiposity (8). A key contributor is the accumulation of SASP, which releases pro-inflammatory cytokines, including IL-1β, IL-1α, IL-6, IL-8, TNF-α, and TGF-β (21). Additionally, damage-associated molecular patterns (DAMPs), which serve as inflammatory stressors, are released from damaged or dead cells and recognized by toll-like receptors (TLRs) on innate immune cells (22). Chronic exposure to inflammatory stressors contributes to the development of innate immune memory and primes the adaptive immune system, ultimately leading to profound age-related immune alterations, which is immunosenescence (23).

Additional contributors to inflammaging include mitochondrial dysfunction (24) and gut dysbiosis (25). Mitochondria serve as a central hub linking oxidative stress, inflammation, and aging. Mitochondrial dysfunction arises from disruptions in redox balance, mitochondrial dynamics, genome stability, and mitophagy, triggering a cascade of cellular disturbances. Age-related mitochondrial dysfunction leads to the increased release of ROS, mitochondrial DNA (mtDNA), and mitochondrial RNA (mtRNA), which activate inflammasomes and act as DAMPs, triggering inflammatory pathways (24). Similarly, gut dysbiosis plays a pivotal role in the development and maintenance of inflammaging (25). The composition and diversity of the gut microbiota are altered with aging, characterized by an increase in pro-inflammatory bacteria, such as Proteobacteria and Enterobacteriaceae, and a reduction in beneficial taxa, including *Bifidobacterium* and *Lactobacillus.* certain anti-inflammatory *Firmicutes* were found to be reduced in the elderly population (26, 27). The impact of dysbiosis is mediated through various immune mechanisms, including compromised intestinal barrier integrity, which allows the translocation of microbial products, such as lipopolysaccharides (LPS), into the circulation, thereby stimulating an immune response (25). Altogether, these mechanisms drive an imbalance between immune regulation and the maintenance of a pro-inflammatory state.

The chronic inflammation characteristic of inflammaging not only drives the pathogenesis of age-related diseases but also plays a central role in the development of frailty (28). Elevated levels of pro-inflammatory cytokines, particularly IL-6 and TNF-α, are consistently associated with the onset and progression of age-related diseases, including cardiovascular disease, type 2 diabetes, and neurodegeneration (28).

Inflammaging, a chronic low-level inflammation associated with aging, contributes to sarcopenia, loss of muscle mass and function, and frailty. This inflammation also drives neuroinflammation, which is linked to cognitive decline. The chronic inflammatory state accelerates functional decline and vulnerability to stress, worsening health outcomes in older adults. Targeting inflammation through therapies like clearing senescent cells, restoring gut microbiota, and improving mitochondrial function may reduce frailty and support healthy aging (29).

### 3.3 Immunometabolism: the overlooked link between intermittent fasting, immune aging, and frailty

One of the least discussed but most consequential features of immune aging is a progressive loss of metabolic flexibility. The ability of immune cells to toggle among glycolysis, fatty acid oxidation (FAO), and mitochondrial respiration underlies their capacity to proliferate, secrete cytokines, and return to homeostasis (30). In young or acutely activated T cells and macrophages, glycolysis predominates even in the presence of oxygen (the “Warburg-like” effect), enabling rapid biomass production to fuel effector functions. By contrast, long-lived memory T cells, regulatory T cells, and alternatively activated (M2) macrophages depend more heavily on oxidative phosphorylation and FAO for sustained activity and anti-inflammatory signalling (30, 31). Aging disrupts this choreography: thymic involution reduces the influx of metabolically “fresh” naïve T cells, mitochondrial quality control deteriorates, and senescent immune cells accumulate, with a rigid, glycolysis-biased, or dysfunctional metabolic profile that correlates with low-grade inflammation and impaired pathogen defence (32).

IF imposes a cyclic metabolic switch through repeated alternation between fed and fasted states, thereby inducing adaptive cellular stress resistance and metabolic flexibility. During fasting, glycogen stores are depleted, lipolysis increases, and circulating free fatty acids rise; hepatocytes and, to a lesser extent, astrocytes convert these substrates into ketone bodies, such as β-hydroxybutyrate (BHB). This switch activates nutrient-sensing pathways, AMP-activated protein kinase (AMPK), sirtuin-1 (SIRT1), and peroxisome proliferator-activated receptor-α (PPAR-α), which in turn promote FAO, mitochondrial biogenesis, and autophagy while dampening NF-κB-driven inflammatory transcription (33). BHB itself has been shown to inhibit the NLRP3 (NOD-like Receptor Family Pyrin Domain Containing 3) inflammasome, reducing IL-1β and IL-18 secretion and favouring an anti-inflammatory milieu (34). Through these mechanisms, IF may reprogram immune cells toward a metabolic state more typical of youthful regulatory or memory phenotypes, thereby improving stress resilience.

This metabolic dimension is particularly relevant to frailty and neuroinflammation. Frailty has been associated with mitochondrial dysfunction, impaired fatty acid handling, and anabolic resistance in skeletal muscle and immune cells (35). Systemic low-grade inflammation and altered energy metabolism reinforce each other, driving sarcopenia and reduced physiological reserve. By repeatedly engaging FAO and ketogenesis, IF may improve mitochondrial efficiency and limit the accumulation of lipotoxic intermediates, such as ceramides, that promote systemic and neuroinflammation (36). In animal models, fasting regimens have been shown to restore T-cell metabolic plasticity, reduce ROS production, and enhance autophagic clearance of damaged organelles. These changes coincide with better infection control and improved cognitive performance (37, 38). Table 1 summarizes the main IF regimens and their immune-metabolic effects relevant to aging.

**Table 1:** Comparative overview of major intermittent fasting (IF) regimens and their immune-metabolic effects in the context of aging.

Regimen	Description	Reported immune/Metabolic effects	Underlying mechanisms	Representative evidence
Time-restricted eating (TRE)	Daily food intake confined to a short window (usually 6–10 h)	Improved circadian alignment, enhanced insulin sensitivity, reduction in circulating IL-6 and TNF-α, broader T-cell repertoire	Activation of AMPK and SIRT1, suppression of NF-κB signalling, rise in NAD^+^ and BDNF	(184, 189)
Alternate-day fasting (ADF)	24 h fast alternated with 24 h ad libitum feeding	Induction of autophagy, decline in senescent T-cell pools, improved mitochondrial efficiency, and lower systemic inflammation	Inhibition of mTOR, up-regulation of LC3-II/Beclin-1, reduction in SASP factors	(18, 190)
Periodic fasting (5:2 diet)	Two non-consecutive fasting days each week	Lower LDL cholesterol and insulin, reduction in oxidative stress, improved innate immune activity	Ketone body production (β-hydroxybutyrate), inhibition of NLRP3 inflammasome, histone deacetylase (HDAC) modulation	(34, 77, 191)
Fasting-mimicking diet (FMD)	5-day plant-based, low-calorie, fasting-mimicking diet with controlled macronutrient composition (Day 1: ~1,090 kcal, 10% protein; Days 2–5: ~725 kcal, 9% protein)	Reduction of immunosuppressive monocytes, rise in activated NK and CD8^+^ T cells, improved stem-cell renewal	Lower IGF-1 and PKA activity, activation of FOXO pathways, stimulation of hematopoietic stem cell proliferation	(126)
Ramadan intermittent fasting (RIF)	Dawn-to-sunset fasting with nightly eating	Lower IL-6, IL-1β, TNF-α; favourable lipid shifts (↓ LDL, ↑ HDL); enrichment of beneficial gut taxa; down-regulation of FTO expression	Up-regulation of SIRT1/AMPK, visceral fat reduction, and increased SCFA production	(66, 67)
Prolonged fasting (>48–72 h) (in mice)	Continuous fasting for more than 2–3 days	Strong autophagy induction, clearance of senescent immune cells, restoration of hematopoietic stem cell function	Suppression of mTOR, activation of AMPK, PDK4-mediated metabolic shift, and enhanced ketogenesis	(38, 126)

IL-6, Interleukin-6; TNF-α, tumor necrosis factor-alpha; AMPK, AMP-activated protein kinase; SIRT1; sirtuin 1; NF- NF-κB, Nuclear factor kappa-light-chain-enhancer of activated B cells; NAD +, nicotinamide adenine dinucleotide; BDNF, brain-derived neurotrophic factor; mTOR, mammalian target of rapamycin; LC3-II, microtubule-associated protein 1A/1B-light chain 3; SASP, senescence-associated secretory phenotype; NLRP3, NOD-like Receptor Family Pyrin Domain Containing 3; IGF-1, Insulin-like growth factor 1; PKA, protein kinase A; FOXO, forkhead box transcription factors; FTO, Fat Mass and Obesity-Associated; SCFAs, short-chain fatty acids; and PDK4, pyruvate dehydrogenase kinase 4.

Yet these benefits are not automatic. Older adults often exhibit reduced glycogen stores, slower metabolic switching, and a greater risk of hypoglycemia or sarcopenia during caloric restriction. Data on frailty outcomes under IF are almost non-existent, and some isocaloric IF trials have reported loss of lean mass, raising concern about IF practice in sarcopenic, pre-frail or frail individuals (39). In a randomized trial in lean adults comparing isocaloric ADF with continuous energy restriction, both approaches produced similar weight loss, however weight loss induced by ADF was attributable to comparable losses of fat and fat-free mass (40). Similarly, in adults with overweight and obesity, a randomized TRE regimen without prescribed calorie restriction was associated with a loss of appendicular lean mass, which serves as a caution for populations at risk for sarcopenia (41). Whether the metabolic and immunological advantages of IF observed in younger or healthier populations translate safely to frail elders remains largely untested. Well-designed trials using ex vivo profiling of immune cell metabolism (glycolysis vs. FAO rates, mitochondrial reserve capacity) alongside functional outcomes (vaccine response, muscle strength, cognition) would help determine whether IF can truly rejuvenate immune function in this demographic. Until then, enthusiasm for IF as a broad anti-aging intervention should be tempered with an appreciation of individual variability and potential trade-offs.

## 4 Frailty in the elderly: a clinical outcome of immune aging

### 4.1 Defining and measuring frailty

Frailty definitions may vary, but two main concepts of frailty prevail. Frailty is conceptualized either as a distinct geriatric syndrome or as a state resulting from multiple accumulated health impairments. Either way, frailty is more common with age; it is one of the most clinically relevant manifestations of aging (42, 43). Frailty is a clinically recognized condition characterized by reduced physiological reserves, decreased functional abilities, dependency, comorbidity, mortality, and increased susceptibility to a wide range of adverse health outcomes (44). According to a report encompassing data from 62 countries, the prevalence of frailty among community-living individuals ranged from 11% in those aged 50–59 to 51% in those aged 90 and above (45). The frailty phenotype is a widely used clinical model that defines frailty based on five physical criteria: unintentional weight loss, self-reported exhaustion, weakness (measured by handgrip strength), slow walking speed, and low physical activity (46). Individuals meeting three or more of these criteria are classified as frail, while those with one or two are considered pre-frail. This phenotype emphasizes the physical manifestations of frailty and is instrumental in identifying older adults at increased risk of adverse health outcomes (46).

Besides observable clinical phenotypes, frailty arises at the biological level as a condition characterized by reduced homeostatic resilience. This loss of physiological adaptability reduces the body’s capacity to maintain stability and respond to stressors (47). At the cellular level, unhealthy aging is closely linked to cellular senescence, a process implicated in the pathogenesis of frailty. Multiple biological processes underlie unhealthy aging and frailty, such as immune system dysregulation, referred to as immunosenescence, as a key mechanism (47).

### 4.2 Immunological underpinnings of frailty

Frailty is underpinned by a collection of age-related immune alterations that compromise physiological integrity and resilience. Among these, immunosenescence plays a pivotal role by impairing immune surveillance, adaptive responses, and tissue repair mechanisms (48). In the context of frailty, these immunological deficits contribute to susceptibility to infections, slower recovery from illness, and reduced response to medical interventions, including vaccinations (48).

Closely intertwined with immunosenescence is inflammaging, the chronic, low-grade inflammation that emerges with advancing age. Elevated levels of systemic inflammatory markers, such as IL-6, TNF-α, and CRP, have been consistently associated with frailty and its clinical manifestations, including sarcopenia, cognitive decline, and functional impairment (49). This persistent inflammation disrupts homeostasis across multiple organ systems, reinforcing the cycle of decline that characterizes frailty. Rather than serving as isolated mechanisms, immunosenescence and inflammaging form a synergistic axis of immune dysregulation that accelerates biological aging and undermines the body’s ability to respond to stress (49). These insights underscore the importance of targeting immune dysregulation to enhance resilience in older adults. A summary of the main immunological and inflammatory biomarkers characteristic of aging is provided in Table 2.

**Table 2:** Key biomarkers associated with immunosenescence and inflammaging in older adults.

Biomarker/ marker	Physiological/ Immune role	Age-related alteration	Clinical relevance to frailty and cognitive decline
IL-6 (8)	Central pro-inflammatory cytokine; modulates the acute-phase response	Chronically elevated with age	Linked to sarcopenia, reduced physical function, cognitive decline, and increased mortality
TNF-α (9)	Orchestrates systemic inflammation and immune cell signalling	Persistent low-grade elevation in the elderly	Associated with frailty, insulin resistance, and neuroinflammation
IL-1β/IL-1α (19)	Mediators of inflammasome activation and SASP	Increased secretion from senescent immune and stromal cells	Drives chronic low-grade inflammation and contributes to inflammaging
CRP (29)	Acute-phase reactant produced by the liver	Mild but consistent elevation with advancing age	Predictor of functional decline, multimorbidity, and poor vaccine response
Serum amyloid A, fibrinogen (20)	Acute-phase proteins reflecting systemic inflammation	Increased baseline levels in older individuals	Associated with cardiovascular risk, frailty, and impaired resilience
CD4^+^CD28^−^ T cells (18)	Senescent T-cell phenotype lacking the co-stimulatory molecule CD28	Expansion with age, reduced diversity of naïve T cells	Poor vaccine response, impaired antiviral defence, and higher frailty scores
CD8^+^CD28^−^/ CD57^+^ T cells (192)	Terminally differentiated cytotoxic T cells	Accumulation with age	Pro-inflammatory SASP secretion, immune exhaustion, vulnerability to infection
B-cell repertoire (48)	Antibody production and immune memory	Skewing toward memory phenotypes, reduced diversity	Impaired vaccine efficacy, increased autoantibody production
NLRP3 inflammasome (61)	Intracellular immune sensor driving IL-1β/IL-18 release	Increased priming and activity in aging	Contributes to systemic inflammaging and neuroinflammation

### 4.3 Immune resilience and recovery potential in frail individuals

Immune resilience is essential to a comprehensive understanding of immunosenescence. It refers to the ability of the immune system to respond effectively to physiological stressors and return to homeostasis, thereby enhancing disease resistance and regulating inflammation during infections and other inflammatory stressors (50). It is notably impaired in frail individuals, as aging is associated with a progressive decline in immune function. This impairment compromises the capacity of immune recovery following infections, injury, or other stressors, contributing to increased morbidity and mortality in older adults (51).

Frail individuals exhibit a reduced ability to elicit effective innate and adaptive immune responses. This is reflected in delayed wound healing, reduced vaccine responsiveness, and prolonged recovery times from acute illnesses. Several immune alterations characterize this compromised resilience, including T-cell exhaustion, persistent inflammaging, and impaired resolution of inflammatory responses effectively (51).

### 4.4 Intermittent fasting and frailty pathways

Although frailty is defined clinically by phenotypes such as unintentional weight loss, exhaustion, reduced grip strength, and slow gait, its biological roots extend into muscle metabolism, oxidative balance, and chronic inflammation. IF may influence each of these pathways in ways that are potentially beneficial but still insufficiently studied in older adults (33). By imposing regular periods of energy deficit, IF enhances lipolysis and ketone body production while activating nutrient-sensing pathways such as AMPK and SIRT1 (33).

The cyclic metabolic “switch” induced by IF may improve insulin sensitivity and anabolic signaling in skeletal muscle, thereby counteracting the blunted protein synthesis that typifies sarcopenic aging (33). Reduced inflammatory tone also benefits neuromuscular function, potentially mitigating frailty-related declines in coordination and resilience (52). Nevertheless, it is important to acknowledge that these mechanisms are derived largely from younger or non-frail populations, and the balance between beneficial stress and nutritional risk in frail elders remains to be determined. Well-designed trials that track muscle mass, strength, inflammatory biomarkers, and functional outcomes are needed to clarify whether IF can be safely integrated into geriatric care as a strategy to delay or reverse frailty.

## 5 Neuroinflammation and neuroplasticity

### 5.1 Role of neuroinflammation in aging and cognitive decline

Another characteristic of aging is a gradual decline in cognitive function, which can result in memory impairment. A critical contributor to age-related cognitive deterioration is neuroinflammation, an inflammatory response of the central nervous system (CNS) to factors that disrupt its homeostasis (53). This impairment of the inflammatory response is characteristic of brain neurodegenerative processes and a key factor in the age-related reduction of neuroplasticity, thereby initiating the pathogenesis of neurodegenerative disorders. Notably, neuroinflammation and oxidative stress can stimulate one another, particularly in the context of illness (53).

Aging inherently promotes neuroinflammation, a state of chronic, low-grade inflammation within the brain (54). This pro-inflammatory environment is driven by the accumulation of ROS and senescent cells that secrete SASP and the release of DAMPs from dying or injured cells (54). Microglia and astrocytes, the two main types of glial cells, undergo noticeable changes in aging; microglia cells are de-ramified, unlike in a healthy brain, where they are characterized by long branches extending from the cell body, to monitor the neuronal microenvironment (54), which leads to higher expression of pro-inflammatory surface markers such as Major Histocompatibility Complex II (MHC II), cluster of differentiation (CD)11b, CD86, and CD68 (55). Astrocytes also exhibit morphological changes and an increase in the expression of the inflammatory surface marker glial fibrillary acidic protein (GFAP) (55). Furthermore, aging alters the brain’s cytokine profile, increasing pro-inflammatory cytokines, such as IL-1β and IL-6, and reducing anti-inflammatory cytokines, including IL-10 and IL-4 (55). These collective cellular and molecular changes result in a chronic state of neuroinflammation and a “primed” glial phenotype, making these cells more prone to robust inflammatory reactions upon stimulation (56). Consequently, aging in the brain demonstrates increased vulnerability to acute inflammatory stimuli, which can lead to more adverse outcomes, even from conditions that are benign in younger individuals (54).

In a study conducted in Germany, researchers developed an obese-aged mouse model. They found that obesity accelerates age-related cognitive decline, accompanied by neuroinflammation, blood–brain barrier disruption, and elevated expression of Spp1, suggesting its potential as an early biomarker for neurodegenerative disorders (57). Another study in mice found that necroptosis, a cell death pathway, increases with age and drives neuroinflammation by activating microglia. Inhibiting this pathway reduced brain inflammation, suggesting necroptosis contributes to age-related cognitive decline (58). Neuroinflammation has also been linked to increased neuronal expression of cathepsin S (CTSS), which activates microglia and promotes a pro-inflammatory environment through the CX3CL1–CX3CR1 and JAK2–STAT3 pathways. Elevated CTSS levels correlate with cognitive decline in both aged mice and Alzheimer’s patients, highlighting its potential role as a biomarker and mediator of age-related neuroinflammation (59).

Additionally, age-related neuroinflammation contributes to disruptions in synaptic homeostasis, impaired hippocampal neurogenesis, and dysregulated neuronal-microglial crosstalk, all of which are linked to a decline in cognitive function (60). Aged microglia are associated with activation of the NLRP3 inflammasome, leading to increased production of IL-1β (61). The activation is driven by accumulated cellular stress, mitochondrial dysfunction, and elevated DAMPs (61). Furthermore, neuroinflammatory mediators can interfere with long-term potentiation (LTP), a key process underlying learning and memory, suggesting a direct mechanistic link between glial activation and synaptic dysfunction (62).

### 5.2 Mechanisms through which intermittent fasting may enhance neuroplasticity

The metabolic switch triggered by IF leads to the release of free fatty acids (FFAs) into the bloodstream, which is then transported to the liver, where it undergoes β-oxidation to generate acetyl-CoA, a precursor for the synthesis of ketone bodies: acetone, acetoacetate (AcAc), and BHB. These ketones cross the blood–brain barrier via monocarboxylate transporters (MCTs), located in the membranes of endothelial cells and neurons. In neurons, BHB and AcAc are converted to acetyl-CoA, which enters the tricarboxylic acid (TCA) cycle in the mitochondria, thereby producing ATP and generating reducing equivalents for the electron transport chain. In addition to circulating ketones, astrocytes can generate ketones, providing a local source of BHB that further supports neuronal energy demands (33, 63). The cyclic metabolic switch induced by IF integrates mitochondrial adaptations, ketone signaling, and activation of nutrient-sensing pathways, including AMPK and SIRT1, thereby exerting neuroprotective effects (64). Recent mechanistic hypotheses suggest that the benefits of metabolic switching may extend beyond neuroprotection to systemic defense mechanisms. It is proposed that the switch from a glucose-dependent state to a fat-dependent, ketogenic state could theoretically suppress viral replication and reduce inflammation and oxidative stress in the context of SARS-CoV-2 infection (65). This conceptual model reinforces the notion that IF-driven metabolic switching induces a systemic adaptive response that simultaneously protects neural tissue and enhances host defense via immunometabolic control. Importantly, the authors emphasize that this is a conceptual model rather than an experimental finding.

In addition to serving as an energy substrate, BHB also stimulates the expression of brain-derived neurotrophic factor (BDNF), which may support mitochondrial biogenesis, synaptic remodeling, and cellular resilience to stress. Additionally, BHB exerts anti-inflammatory effects, modulating neuroinflammation by inhibiting pro-inflammatory cytokines such as IL-1β, IL-6, and TNF-α, which can impair hippocampal synaptic plasticity, providing a possible mechanism underlying inflammation-induced cognitive impairment (60).

Another important class of metabolites influenced by IF is short-chain fatty acids (SCFAs), such as butyrate, propionate, and acetate, which may modulate the gut-brain axis and affect neuroinflammation (37). IF has been shown to influence gut microbial composition and diversity, enhancing alpha diversity and increasing the abundance of bacterial taxa with anti-inflammatory properties (66). IF has been linked to higher concentrations of *Akkermansia muciniphila*, *Lactobacillus*, *Faecalibacterium prausnitzii*, and *Bifidobacterium longum* (67, 68). These beneficial bacteria produce metabolites that support mucosal immunity and suppress pro-inflammatory pathways by inhibiting NF-κB activation (48) and by inhibiting NLRP3 inflammasome formation, which is highly active in microglia during neuroinflammation (69).

In parallel, IF reduces circulating ceramide levels, bioactive sphingolipids known to impair mitochondrial respiration and promote neuroinflammation (36). RIF model has been shown to significantly reduce plasma sphingolipid and ceramide profiles in adults with obesity (70). Elevated ceramides are associated with increased ROS, impaired ATP production, and enhanced neurodegenerative signaling. IF mitigates ceramide-induced mitochondrial dysfunction and enhances neuronal metabolic flexibility, thereby reducing oxidative and inflammatory stress in the brain (36).

Intermittent metabolic switching also improves insulin sensitivity, enhancing neuronal glucose uptake and utilization. Upon refeeding, glucose and dietary carbohydrates stimulate the release of the incretin hormone glucagon-like peptide 1 (GLP-1) from gut enteroendocrine cells into the bloodstream. GLP-1 facilitates glucose clearance by stimulating insulin secretion and increasing insulin sensitivity (33). This aligns with meta-analytic evidence showing that IF significantly reduces insulin sensitivity biomarkers, such as HOMA-IR (71). Notably, GLP-1 can cross the blood–brain barrier and act directly on neurons to support synaptic function, cognitive performance, and cellular stress resistance (33).

**Figure 2:**
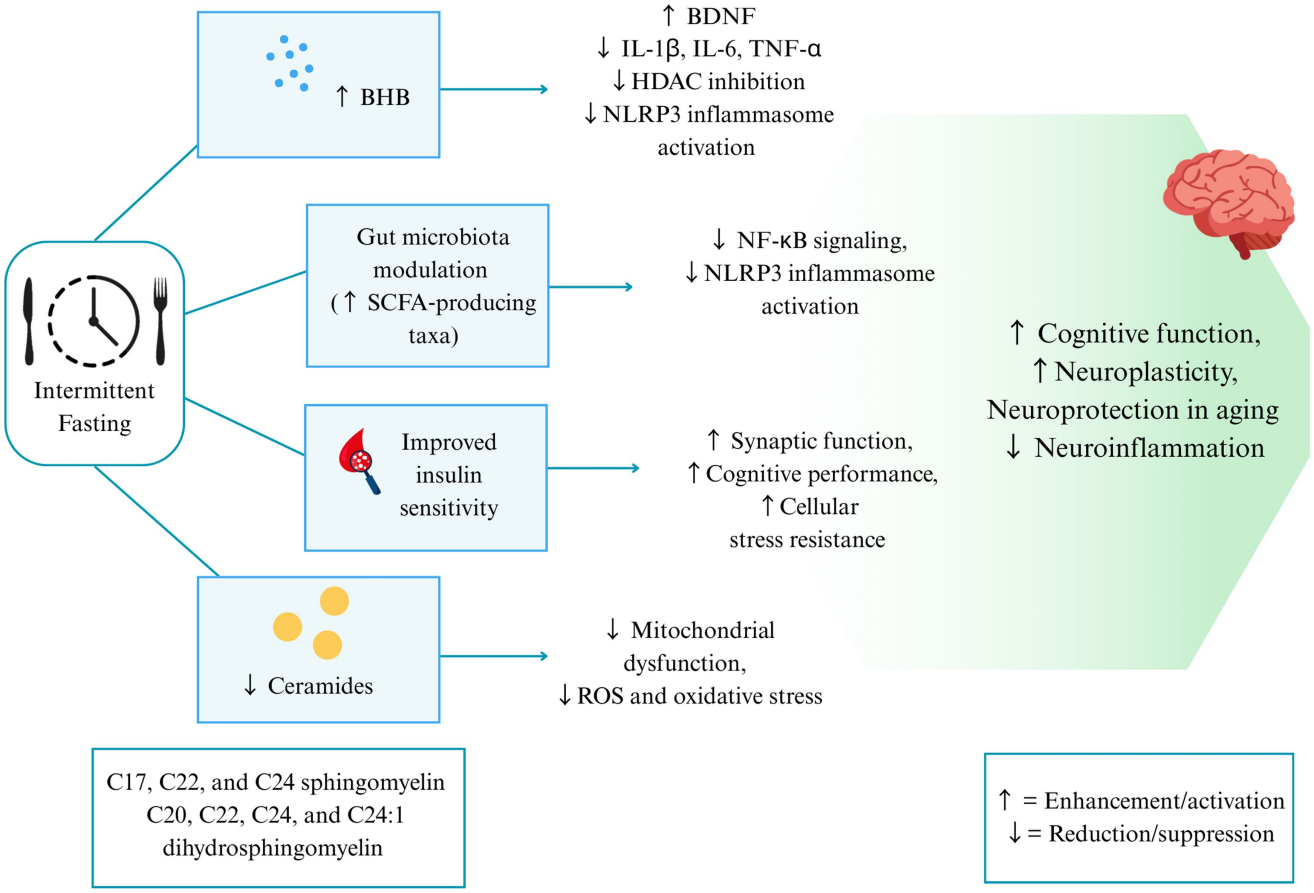
Effects of intermittent fasting on neuroinflammation and brain health. Intermittent fasting (IF) is associated with fasting-related metabolic adaptations, including periodic increases in circulating β-hydroxybutyrate (BHB), reflecting a shift toward fatty acid oxidation during periods of reduced energy and carbohydrate intake. IF may also modulate gut microbiota composition, including enrichment of short-chain fatty acid (SCFA)-producing taxa, with effects dependent on dietary intake and microbial composition. IF has been associated with improvements in insulin sensitivity markers such as fasting insulin and HOMA-IR. IF is also linked to reductions in ceramides and related sphingolipids. Collectively these changes modulate inflammatory pathways (↑ brain-derived neurotrophic factor [BDNF]; ↓ interleukin-1β [IL-1β], interleukin-6 [IL-6], tumor necrosis factor-α [TNF-α]; histone deacetylase [HDAC] inhibition; ↓ NOD-like receptor family pyrin domain containing 3 [NLRP3] inflammasome and nuclear factor κB [NF-κB] signaling; ↓ mitochondrial dysfunction, reactive oxygen species [ROS] and oxidative stress), leading to improved synaptic function, cognitive performance, cellular stress resistance, neuroplasticity, and neuroprotection with reduced neuroinflammation. BHB, β-hydroxybutyrate; SCFAs, short-chain fatty acids; BDNF, brain-derived neurotrophic factor; IL-1β, interleukin-1 beta; IL-6, interleukin-6; TNF-α, tumor necrosis factor-alpha; HDAC, histone deacetylase; NLRP3, NOD-like receptor family pyrin domain containing 3; NF-κB, nuclear factor kappa-light-chain-enhancer of activated B cells; ROS, reactive oxygen species.

Moreover, BHB has been shown to act as an epigenetic regulator by inhibiting class I histone deacetylases (HDACs), which are inhibitors of the NLRP3 inflammasome, reducing neuroinflammatory cytokine release, and preserving neuronal integrity. Inhibition of HDACs also increases the expression of neuroprotective genes, such as BDNF, which contributes to enhanced synaptic plasticity, particularly in brain regions vulnerable to aging, like the hippocampus, and improves cognitive resilience (72, 73). Figure 2 illustrates the mechanisms through which IF affects neuroinflammation.

### 5.3 Potential cognitive benefits in older adults

Recent evidence suggests that IF may offer meaningful cognitive benefits for older adults, particularly those with insulin resistance or obesity. A recent systematic review highlights the diverse study designs, ranging from cross-sectional to experimental. It suggests a relationship between TRE, IF, cognitive function, and mental health among older adults. For instance, older adults practicing TRE, especially those over 70, were less likely to exhibit symptoms of mental health distress (74). A pilot study in older adults with self-reported memory decline found that an 8-week intermittent intervention of prolonged nightly fasting of 14 h per night, without any dietary restrictions during the refeeding period, significantly improved neurocognitive function (75). Similarly, a 36-month study in older Malay adults with mild cognitive impairment found that those who practiced dry IF from dawn to dusk two days a week regularly showed significant improvements in cognitive function, reduced inflammation, oxidative stress markers, and favorable metabolic changes, with 24.3% reverting to successful aging, which is higher than irregular fasters or non-fasters (76).

Experimental and cohort studies further support the feasibility of fasting interventions, though findings related to mood and anxiety are mixed. Notably, TRE has demonstrated potential neuroprotective effects, including reductions in neuroinflammatory markers linked to cognitive decline and Alzheimer’s disease. The benefits of TRE appear to depend not only on fasting duration but also on meal timing; consuming meals earlier in the day has been linked to improvement in metabolic markers, such as insulin resistance, inflammation, and lipid profiles, which are associated with cognitive function (74). A pilot study involving participants aged 65 and above with overweight found that a 16:8 fasting protocol for 4 weeks, with no calorie restriction during the intervention, did not result in significant improvements in cognitive function or quality of life (77). In contrast, two cross-sectional studies in an Italian cohort reported that individuals practicing 8- or 10-h TRE had a reduced likelihood of cognitive impairment and mental distress, particularly among those older than 70 (78, 79). Longitudinal data demonstrated that older adults aged 60 years and above with mild cognitive impairment, practicing regular dry IF from dawn to dusk two days a week, over 36 months, were associated with improved cognitive performance, higher rates of successful aging, and favorable changes in biomarkers, including increased antioxidant activity and reduced inflammation (80). Similarly, older adults who remained physically active during Ramadan fasting exhibited better executive function and memory compared to sedentary individuals (81). Collectively, these findings suggest that IF may benefit cognitive and emotional health in aging populations. Still, responses may vary depending on individual factors and the specific fasting protocol applied.

In addition to findings in cognitively healthy populations, evidence from Alzheimer’s disease research further supports the neuroprotective potential of IF. According to a recent review, TRE may reduce beta-amyloid 42 (Aβ42) deposition and pro-inflammatory cytokines, while also improving gut microbiota composition and regulating the circadian rhythm, factors closely linked to cognitive function and aging. These mechanistic changes may contribute to slower disease progression and suggest a broader relevance of TRE for cognitive health in aging populations (82).

However, a large, randomized trial of continuous energy restriction has yielded less favorable results. The look AHEAD trial in adults with type 2 diabetes, and overweight or obesity, tested an intensive lifestyle interventions emphasizing on continuous energy restriction and weight loss in adults with type 2 diabetes, following an intensive lifestyle program emphasizing daily caloric restriction and increased physical activity for an average of 10 years, did not reduce the risk of cognitive impairment compared with standard care over long-term follow up (83). In a secondary analysis of the same trial, greater weight reduction in individuals with obesity was associated with a stronger decline in selected cognitive domains, raising concern that aggressive or prolonged energy restriction may adversely affect cognition in vulnerable individuals (84). Although these studies do not directly test intermittent fasting, the findings suggest that energy restriction and weight loss have mixed effects on cognition and may even be detrimental in some individuals, a consideration when applying fasting-based strategies to older or high-risk populations.

## 6 Interactions between intermittent fasting, inflammaging, and immunosenescence

Emerging evidence suggests that IF may counteract key drivers of inflammaging and immunosenescence through diverse molecular and cellular mechanisms, including inflammatory signaling, enhanced autophagy, and activation of adaptive stress-response pathways.

### 6.1 Cellular stress pathways and inflammatory modulation in intermittent fasting

IF triggers a series of adaptive cellular responses that enhance resilience against metabolic and oxidative stress. One of the key mechanisms involves the activation of cellular stress sensors, including SIRT1 and the antioxidant genes nuclear factor erythroid 2-related factor 2 (NRF2), mitochondrial transcription factor A (TFAM), and superoxide dismutase 2 (SOD2) (85, 86). SIRT1, a NAD-dependent deacetylase, modulates inflammation by deacetylating transcription factors such as NF-κB, thereby reducing the expression of pro-inflammatory cytokines, including IL-6 and TNF-α. This process protects cells from oxidative damage and suppresses inflammatory signaling (85). Human trials have investigated the effects of RIF on pro-inflammatory cytokines, revealing significant anti-inflammatory effects. In overweight and obese adults, RIF led to notable reductions in IL-6, TNF-α, C-reactive protein (CRP), and high-sensitivity CRP (hs-CRP), along with improved lipidomic profiles and decreased sphingolipid species associated with inflammation (70, 87–89). However, other evidence suggests heterogeneous inflammatory responses to IF. A systematic review and meta-analysis of randomized controlled trials in individuals with obesity/overweight found that IF significantly reduced TNF-α, but had no significant effect on CRP or IL-6. In contrast, CR significantly reduced CRP and IL-6, but not TNF-α (90). Another meta-analysis of IF interventions reported a significant reduction in TNF-α and leptin, but not IL-6 and adiponectin (91). The inflammatory effects of IF were inconsistent across fasting protocols. (92, 93) Taken together, these findings suggest that IF may modestly improve certain inflammatory markers in specific settings, but overall anti-inflammatory effects in humans are small, heterogeneous, and highly dependent on the fasting regimen, habitual diet, and study population.

### 6.2 Enhanced autophagy and cellular resistance

IF may influence the immune system by promoting autophagy. Under conditions of energy deprivation, cells break down damaged organelles to conserve energy (94, 95). This process of “cellular fasting” or “cellular famine” initiates the hydrolysis of triglycerides into fatty acids and triggers autophagy. Autophagy involves the encapsulation of proteins and organelles within autophagosomes, which then fuse with lysosomes to break down their contents, providing the cell with a source of energy (96). Numerous studies have suggested that IF induces autophagy. Early-TRE for four days in humans, with overweight and energy provided to meet weight-maintenance requirements under sedentary conditions, resulted in increased levels of SIRT1 and the autophagy-related gene microtubule-associated protein one light chain 3A (LC3A) in blood (97). Additionally, a recent human study using the RIF model, which involves dawn-to-dusk intermittent fasting, demonstrated a significant upregulation of autophagy markers and an improved inflammatory profile in an overweight and obese cohort, providing direct evidence for IF’s immune benefits in humans (98).

Autophagy plays a crucial role in clearing damaged proteins and organelles, while supporting mitochondrial function, as mitochondria serve as a key site for autophagic vesicles (99). Autophagy’s role in cytoplasmic clean-up is inherently anti-inflammatory (100). Autophagy also contributes to immunometabolic states, influencing macrophage and T cell polarization (101). Additionally, autophagy serves as an antimicrobial defense against intracellular pathogens such as *Mycobacterium tuberculosis* (102) and *Streptococcus* (103). AMPK is linked with anti-inflammatory activity and metabolic quiescence, while mTOR supports inflammation and a robust immune response (104). Animal studies have shown that TRF, long-term fasting, or refeeding after fasting in mice activate AMPK (105, 106). In parallel, AMPK suppresses the mammalian target of rapamycin (mTOR), a known inhibitor of autophagy. Fasting-associated downregulation of mTOR has been observed in mice (105, 106) and even in organisms like *Schmidtea mediterranea* (107). Collectively, the fasting-induced activation of AMPK and inhibition of mTOR suggest that IF may exert an anti-inflammatory impact. AMPK also supports T cell differentiation, enhancing the responses of Th1 and Th17 cells and the functionality of Treg cells (108).

### 6.3 Metabolic-immune crosstalk during intermittent fasting-induced delayed aging

During prolonged fasting, energy metabolism shifts from glucose dependence toward fatty-acid driven ketone production, releasing FFAs and glycerol into circulation, and resulting in ketogenesis (109). These FFAs not only serve as energy substrates but also play a crucial role in obesity-induced adipose tissue inflammation (58), immunomodulation (59), and the activation of hepatic very low-density lipoprotein (VLDL) production (110). This metabolic switch is particularly relevant in the context of aging, as chronic inflammation is a key contributor to both immunosenescence and inflammaging (19). Consistent with cyclic metabolic switch theory, as mentioned earlier, the repeated transition between fed and fasting states could enhance long-term cellular resilience to stress, promote plasticity, cognition, and reduce inflammation, contributing to improved immune function and metabolic health (64).

While saturated FFAs promote inflammation by activating macrophages (111). Elevated FFAs during fasting activate nuclear receptors, including PPAR-*α* and activating transcription factor 4 (ATF4). Resulting in increased production of fibroblast growth factor 21 (FGF21), a hormone that upregulates SIRT1 activity via positive feedback and reduces oxidative stress, thereby mitigating inflammation (112).

As fatty acid oxidation increases in the liver, acetyl-CoA accumulates (113), leading to activated pyruvate dehydrogenase kinase 4 (PDK4) (114). Activated PDK4 inhibits pyruvate dehydrogenase (PDH), a key enzyme in carbohydrate metabolism, and this metabolic regulation has been linked to the modulation of inflammation (114). A study conducted on 17 male subjects found that trained individuals who fasted for 24 h exhibited enhanced regulation of lipolysis, glyceroneogenesis, and substrate availability in adipose tissue compared to fasting untrained individuals, while upregulating expression of PDK4 (115). Also, PDK4 expression was upregulated in skeletal muscle mass in eleven healthy adults who fasted for 40 h (116), further reinforcing this metabolic adaptation.

Moreover, the ketone body BHB, a key product of fasting-induced fat oxidation, also acts as an epigenetic modifier that promotes the expression of the oxidative stress resistance gene, and shows anti-oxidative stress effects in mice (117). As detailed in section 3.3, BHB can modulate inflammasome signaling (34) and activate the hydroxycarboxylic acid receptor 2 (HCA2), which is implicated in metabolism and innate immunity (118). Notably, these antioxidant effects contribute to reduced accumulation of senescent cells and inflammatory mediators (19). Furthermore, IF has been shown to increase nicotinamide adenine dinucleotide (NAD+) levels, as evidenced in mice that underwent ADF for 1 month (119). NAD + is an essential cofactor for sitruin-mediated regulation of inflammation in both immune and non-immune cells (120). Altogether, mechanistic data from animal models and small human studies support plausible anti-inflammatory pathways activated during fasting. Still, human evidence for sustained reductions in systemic inflammatory burden remains heterogeneous and inconclusive, partly because fasting is timed differently, involves different caloric restrictions, and uses different dietary schemes.

### 6.4 The mTOR–T-cell aging axis and intermittent fasting

Among the nutrient-sensing pathways implicated in immune aging, the mTOR occupies a central position. mTOR integrates signals from amino acids, glucose, growth factors, and cytokines to regulate cell growth, metabolism, and survival. mTOR signaling is not restricted to the cytoplasm; mTOR complexes also localize to the nucleus, where they interact with transcriptional and chromatin-remodeling machinery to orchestrate gene programs that coordinate cellular growth, metabolism, and stress responses. Through these nuclear actions, mTOR couples nutrient availability to long-term changes in cell fate and tissue homeostasis, including aging-related remodeling of immune and stromal compartments (121).

In T cells, sustained mTOR activity promotes glycolytic metabolism and effector differentiation, whereas reduced mTOR signaling favors memory and regulatory phenotypes with enhanced longevity (122). Aging is often associated with chronic, low-grade mTOR activation, contributing to T-cell senescence, diminished naïve T-cell pools, and a skewed CD4/CD8 ratio, hallmarks of immunosenescence (32, 123). In parallel, upregulated mTOR activity in skeletal muscle contributes to mitochondrial dysfunction, impaired proteostasis, and loss of muscle mass and strength, which manifest clinically as sarcopenia and frailty and further undermine immune resilience in older adults (124).

IF can modulate this axis by periodically lowering circulating amino acids, insulin, and IGF-1, thereby reducing mTOR complex 1 (mTORC1) activity and indirectly enhancing AMPK and SIRT1 signaling. These shifts create a metabolic environment that mimics some effects of rapamycin, one of the most robust lifespan-extending interventions in animal models, without pharmacological inhibition (52). Recent work also shows that amino acids regulate blood glucose by engaging mTORC1-dependent signaling in pancreatic *β* cells, modulating transcriptional regulators of insulin production, and thereby linking amino acid sensing to systemic glucose homeostasis. Intermittent fasting–induced oscillations in amino acid availability may thus reprogram mTOR signaling across immune cells, muscle, and metabolic organs, coordinating metabolic adaptation with preservation of immune homeostasis during aging (125).

In preclinical studies, fasting or fasting-mimicking diets have increased the proportion of naïve and central memory T cells, improved vaccine responses, and reduced inflammatory cytokine production (38, 126). Such findings suggest that cyclic dampening of mTOR activity may help rejuvenate the T-cell compartment, enhance immune resilience, and potentially slow immunosenescence in older adults.

However, the balance is delicate: excessive suppression of mTOR can impair effector responses required to combat acute infection or malignancy. Whether intermittent nutritional modulation can achieve a “sweet spot” of mTOR activity sufficient to maintain immune vigilance while promoting longevity-associated phenotypes remains to be determined in well-designed clinical trials focused on aged and frail populations.

**Figure 3:**
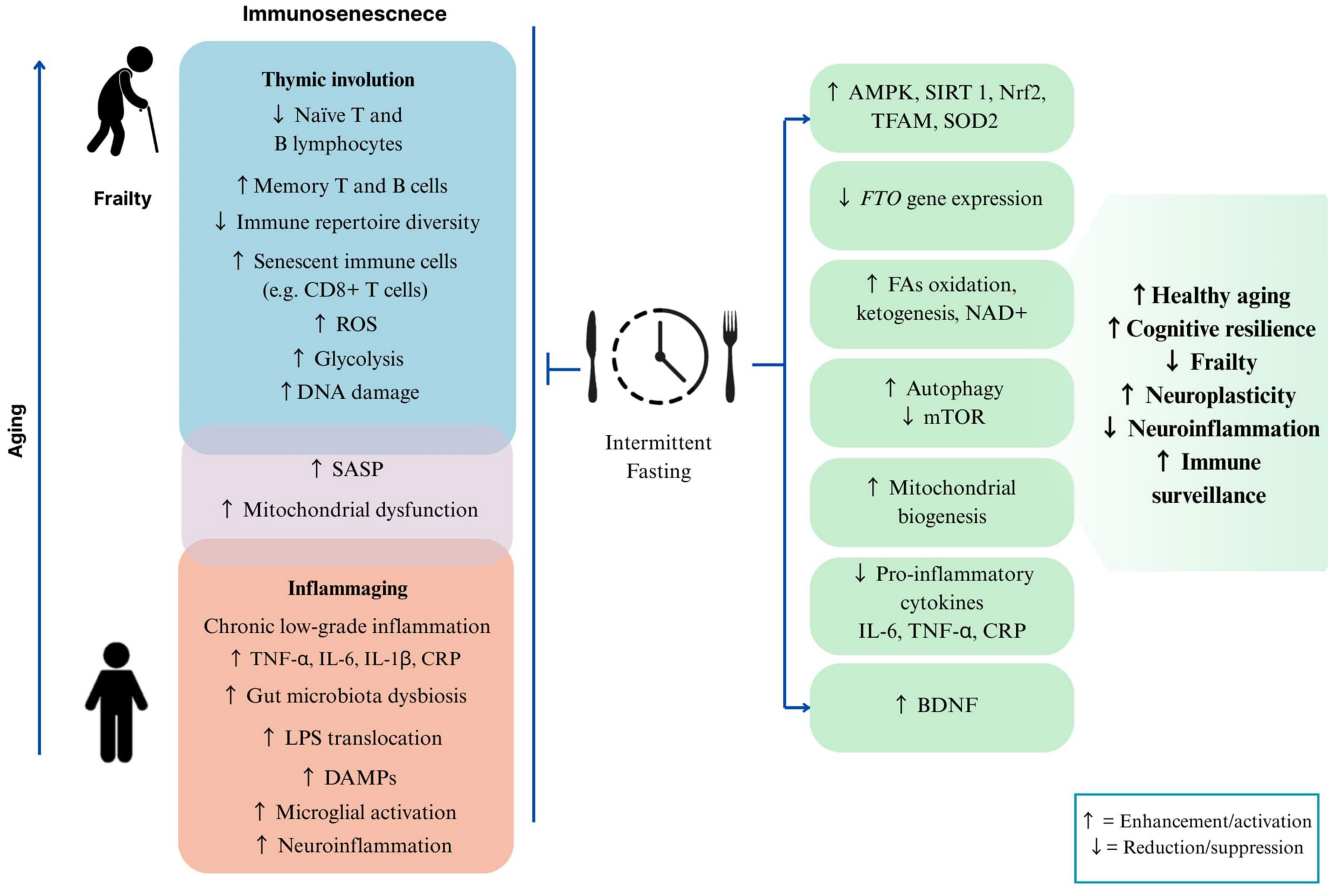
Effects of intermittent fasting on immunosenescence, inflammaging, and frailty. Immunosenescence and inflammaging accompany aging and are characterized by thymic involution, loss of naïve T and B lymphocytes, accumulation of senescent immune cells, increased glycolysis, DNA damage, mitochondrial dysfunction, and a senescence-associated secretory phenotype (SASP), along with low-grade inflammation, gut microbiota dysbiosis, lipopolysaccharide (LPS) translocation, damage-associated molecular patterns (DAMPs), microglial activation, and neuroinflammation. Intermittent fasting (IF) counters these processes by activating AMP-activated protein kinase (AMPK), sirtuin-1 (SIRT1), nuclear factor erythroid 2-related factor 2 (Nrf2), mitochondrial transcription factor A (TFAM), and superoxide dismutase 2 (SOD2); reducing fat mass and obesity-associated (FTO) gene expression and mammalian target of rapamycin (mTOR) activity; promoting fatty acid oxidation, ketogenesis, nicotinamide adenine dinucleotide (NAD^+^) availability, autophagy, mitochondrial biogenesis, and brain-derived neurotrophic factor (BDNF); and lowering pro-inflammatory cytokines (IL-6, TNF-α, IL-1β) and C-reactive protein (CRP). These adaptations support healthy aging, cognitive resilience, increased neuroplasticity and immune surveillance, and reduced frailty and neuroinflammation. Abbreviations: ROS, reactive oxygen species; SASP, senescence-associated secretory phenotype; TNF-α, tumor necrosis factor-alpha; IL-6, interleukin-6; IL-1β, interleukin-1 beta; CRP, C-reactive protein; LPS, lipopolysaccharides; DAMPs, damage-associated molecular patterns; AMPK, AMP-activated protein kinase; SIRT 1, sirtuin-1; Nrf2, nuclear factor erythroid 2-related factor 2; TFAM, mitochondrial transcription factor A; SOD2, superoxide dismutase 2; FTO, fat mass and obesity-associated; mTOR, mammalian target of rapamycin; BDNF, brain-derived neurotrophic factor.

The proposed mechanisms by which IF influence immunosenescence and inflammaging, and their implications for aging and frailty, are illustrated in Figure 3.

### 6.5 Role of the oral-gut microbiota axis in frailty

Aging is associated with a decline in oral microbial diversity, including a reduction in beneficial bacteria and a rise in potentially harmful species, which can lead to gum disease, dry mouth, and tooth decay (127). *Neisseria* levels generally decrease after the age of 40, whereas levels of *Lactobacillaceae*, *Streptococcus anginosus*, and *Gemella sanguinis* tend to increase after the age of 60 (128).

The gut microbiota has long overshadowed the oral microbiota in research. However, interest in the oral microbiome has surged in recent years (129, 130), placing it among the top five priorities of the Human Microbiome Project (131). Despite extensive focus on the gut microbiota and its well-established impact on digestion, immunity, and overall health, the oral microbiota also significantly influences oral and systemic health, as it is the body’s first interface with ingested substances (129, 132). Both oral and gut microbiota alterations lead to inflammaging and mitochondrial dysfunction, which are hallmarks of aging (117) and the underlying mechanisms of frailty and sarcopenia (133). Emerging evidence links the oral-gut microbiota axis to age-related outcomes, including frailty and sarcopenia (134).

The connection between the gut and the oral microbiota is an emerging field that has been gaining attention, referred to as the oral-gut microbiota axis (135). Given their anatomical continuity as parts of the gastrointestinal tract, microbes from the oral cavity can migrate and colonize in the gut (136). Recently, 61 shared amplicon sequence variants (ASVs) were found in the gut and oral microbiota in 96% of the participants studied (137). Of these, 26 variants were found in children and adults, suggesting lasting colonization (137). Communication between the oral and gut microbiota occurs through the complex, bidirectional gut-oral axis. The bidirectional communication occurs through various pathways, such as the bloodstream, saliva, and fecal-oral routes (136).

The enteral route is through the swallowed saliva; around 1–1.5 liters of saliva are ingested daily, transporting microbes into the gastrointestinal tract (138). Although gastric acid and bile acids typically eliminate microbes, certain factors, such as infancy, aging, gastrointestinal diseases, and the use of medications like antibiotics and proton pump inhibitors, can impair these defenses (136, 138, 139), allowing pathogens like *Porphyromonas gingivalis*, *Klebsiella* spp., *Helicobacter pylori*, *Streptococcus* spp., *Veillonella* spp., *Parvimonas micra*, and *Fusobacterium nucleatum* that can survive acidic environments to colonize in the gut (140, 141). Concurrently, microbes may also enter the bloodstream through mechanical actions, such as chewing, brushing, or dental procedures, particularly when periodontal tissues are inflamed (138, 140). Once in the bloodstream, these microbes can reach the gut, disrupt the intestinal barrier, and trigger systemic inflammation (138, 140). Additionally, the fecal-oral route enables the bidirectional transfer of microbes, particularly in conditions of poor hygiene or among immunocompromised individuals (136, 142). Contaminated hands, food, or water may facilitate the migration of gut microbes back to the oral cavity, potentially worsening oral dysbiosis and promoting pathogenic colonization (142). These pathways underscore the dynamic nature of the oral-gut microbiota axis.

Disruptions of the oral-gut microbiota axis have been increasingly implicated in frailty, particularly through their shared contributions to chronic low-grade inflammation, mitochondrial dysfunction, and oxidative stress (134). Oral pathogens such as *P. gingivalis* and its LPS can translocate to the gut, compromising intestinal barrier integrity and promoting metabolic endotoxemia by upregulation of toll-like receptors 2 (TLR2), TNF-α, and IL-17 (140). This occurs via *P. gingivalis* downregulating tight junction proteins zonula occludens 1 (ZO-1) and occludin in the small intestines (143), besides increased LPS in the bloodstream that leads to upregulation of flavin-containing dimethylaniline monooxygenase three expression (FMO3) and increases concentrations of circulating trimethylamine N-oxide (TMAO), thereby enhancing metabolic dysregulation, gut dysbiosis, inflammation, and intestinal permeability (143, 144).

*P. gingivalis* also promotes IL-6 expression through the Janus kinase 2/glycogen synthase kinase 3-*β*/signal transducer and activator of transcription 3 (JAK2/GSL3-β/STAT3) pathway, which interferes with mitochondrial apoptosis mechanisms, contributing to inflammaging and metabolic dysfunction, which are linked to frailty and physical decline in older adults (145).

Moreover, metabolites such as SCFAs may be translocated from the gut to the oral cavity via the bloodstream, thereby affecting oral pH (146, 147). Additionally, the oral microbiota produces SCFAs through carbohydrate metabolism, similar to the gut microbiota (110), albeit at lower concentrations (148, 149). SCFAs exert anti-inflammatory effects, including reducing ROS production (110), suppressing TNF-α and IL-12, supporting mucosal barrier integrity, and re-establishing immune equilibrium through the Treg/Th17 balance (140, 145, 150). Additionally, SCFAs can potentially affect muscle metabolism (115–117). For example, muscle cells utilize acetate to generate energy (151). Depletion of SCFAs may promote anabolic resistance and muscle catabolism, which is a characteristic of sarcopenia in frail older adults (152–154). Nevertheless, the role of the oral microbiota in maintaining muscle mass remains understudied and warrants further investigation.

Epidemiological studies support the connection between salivary microbiota and frailty; higher abundance of *Actinomyces*, *Streptococcus*, *Bacilli*, *Selenomonas*, *Veillonella*, and *Haemophilus* taxa were found in nursing home residents who are usually characterized by increased frailty, besides decreased *Prevotella*, *Leptotrichia*, *Campylobacter*, and *Fusobacterium* (155). Frailty was also associated with reduced microbiota diversity in a UK cohort of adult twins. Altogether, these findings highlight the role of the oral-gut microbiota axis in the pathogenesis of frailty (156), offering a fresh perspective for early intervention and preventive strategies targeting microbial health.

### 6.6 Microbiome circadian rhythms, time-of-day feeding, and intermittent fasting

The gut microbiome is not static; its composition and metabolic activity oscillate over the 24-h cycle in synchrony with host circadian clocks. In healthy individuals, daytime feeding supports the expansion of taxa specialized in carbohydrate fermentation, while fasting periods allow enrichment of bacteria that metabolize host-derived substrates such as mucins and bile acids (157). These oscillations influence the production of SCFA, secondary bile acids, and microbial metabolites that, in turn, modulate intestinal barrier integrity, systemic inflammation, and immune cell function. Aging and erratic eating patterns can blunt these microbial rhythms, leading to reduced diversity, loss of temporal compartmentalization, and increased susceptibility to metabolic and inflammatory disorders (158).

IF may restore or strengthen these microbial oscillations by imposing regular fasting–feeding cycles aligned with the light–dark schedule. In animal models, time-restricted feeding has been shown to re-establish diurnal fluctuations in microbial composition, enhance production of beneficial metabolites, and improve metabolic and inflammatory markers even without calorie restriction (159). Human observational studies during Ramadan fasting also report shifts in gut taxa consistent with increased SCFA production and improved lipid and glucose profiles (67). Such findings suggest that the timing of food intake is as important as its content in shaping the microbiome–immune axis. By synchronizing microbial and host circadian rhythms, IF could reduce endotoxemia, dampen systemic inflammation, and ultimately support healthier aging trajectories. However, controlled trials in older, frail populations remain scarce.

### 6.7 *FTO* gene regulation

Poor outcomes in elderly individuals, such as higher rates of mortality (160), are linked to both obesity and frailty. Specifically, visceral adiposity may contribute to frailty by promoting inflammation and insulin resistance (161). A systematic review and meta-analysis demonstrated that obesity in older adults is associated with frailty (162). Adipose tissue acts as a metabolically active organ, releasing adipocytokines such as leptin, adiponectin, IL-6, and TNF-α, which contribute to inflammation (149), reduced skeletal muscle mass and strength (150), and sarcopenic obesity (163). Sarcopenic obesity is known to be associated with diminished physical performance and a higher risk of frailty (164, 165). Furthermore, increased visceral adiposity in older adults is associated with an elevated risk of cognitive decline (166). It is also important to note that frailty is a dynamic condition that may promote fat accumulation and sarcopenia (muscle mass wasting) (167). Given the suggested link between obesity and frailty, it is essential to understand the molecular mechanisms underlying adiposity and metabolic dysfunction. Among these, the fat mass and obesity-associated (*FTO*) gene has emerged as a central regulator of energy homeostasis (168).

The *FTO* gene, particularly the rs9939609 A allele, is strongly associated with a higher risk of obesity (168). *FTO* rs9939609 Single Nucleotide Polymorphism (SNP) has been linked to higher intake of macronutrients, especially fat and carbohydrates, as well as overall greater total energy consumption, while showing no effect on energy expenditure (169). Additionally, the *FTO* gene is expressed in the hypothalamic region, specifically in the arcuate nucleus, a key brain region responsible for regulating appetite, suggesting that it may influence obesity through the regulation of appetite and satiety (169).

Emerging evidence suggests that IF can regulate *FTO* gene expression and activity, offering insight into how dietary interventions might modulate this gene’s role in metabolic and inflammatory processes. IF, particularly RIF, was found to downregulate FTO gene expression in a cohort of individuals with overweight and obesity compared to their pre-fasting state after fasting during the month of Ramadan (170). RIF showed a beneficial impact on cardiometabolic risk factors by reducing body weight, body fat mass, and waist circumference, and by increasing HDL and decreasing LDL (170). These findings are consistent with a meta-analysis that substantiated similar effects of IF on reducing total cholesterol, LDL, and triglyceride levels, as well as diastolic blood pressure and heart rate (171).

Moreover, RIF demonstrated a reduction in pro-inflammatory cytokines IL-6 and TNF-α (170). Similarly, another study showed that RIF reduced IL-1β, IL-6, and TNF-α in fasting subjects of both sexes (87). These findings are further supported by clinical evidence, where RIF significantly reduced serum proinflammatory cytokines, including IL-1β, IL-6, and TNF-α, CRP, and *hs*-CRP, along with oxidative stress markers such as malondialdehyde and urinary 15-f(2 t)-isoprostane (89, 172). Notably, these reductions were associated with significant decreases in visceral adiposity among individuals with obesity after fasting during the lunar month of Ramadan, reinforcing the link between reduced inflammation and improved metabolic outcomes (88).

#### 6.7.1 Neuroinflammatory implications of *FTO* gene regulation

Beyond its association with obesity and energy balance, the *FTO* gene’s biological functions extend to the brain. The *FTO* gene is highly expressed in the brain, in regions such as the hypothalamus, hippocampus, and cortex, where it modulates neuronal activity and synaptic plasticity (173). The *FTO* was identified as the first RNA demethylase; it demethylates N6-methyladenosine (m^6^A), the most abundant RNA modification in a cell (174).

Importantly, the impact of *FTO* on brain health may be mediated not only by its neuronal expression but also by systemic metabolic dysregulation. For instance, obesity itself may drive neuroinflammation by releasing pro-inflammatory molecules from excess adipose tissue. Leading to low-grade systemic inflammation (175). This systemic inflammation can compromise the integrity of the blood–brain barrier, and the released cytokines by the adipose tissue and immune cells can cross this barrier and enter the central nervous system, where the microglia are then activated, resulting in changes in their function and morphology, and therefore, may promote neuroinflammation (176). Preclinical evidence indicates that diet-induced obesity is associated with disrupted myelin and increased numbers of activated microglia and reactive astrocytes. These changes may lead to increased release of pro-inflammatory molecules into the circulation and, therefore, promote neuroinflammation (175).

Notably, the *FTO* gene has a multifaceted, context-dependent role. Besides its role in obesity, *FTO* plays an important role in neurogenesis. *FTO* is highly expressed in neural stem cells and differentiated neurons, with dynamic regulation throughout postnatal neurodevelopment (177). *FTO* deficiency reduces brain size and can impair neurogenesis, particularly by decreasing neural stem cell proliferation and neuronal differentiation *in vivo*, thereby affecting learning and memory (177). Moreover, FTO loss led to dysregulated expression of key components of the BDNF signaling pathway (177). Recent evidence also suggests that hippocampal *FTO* deficiency can induce depressive and anxiety-like behaviors, as well as cognitive impairment, in aged mice, mediated by disruptions in BDNF-tropomyosin-related kinase B (TrkB) signaling and synaptic plasticity, indicating an age-dependent neuroprotective role of *FTO* (178). This preclinical evidence suggests that *FTO* deficiency may increase the susceptibility of depression in older adults in an age-dependent manner, which proposes that *FTO* activators may represent a potential therapeutic strategy for treating depression in older adults (178).

Importantly, late-life depression itself has been linked to pro-inflammatory processes, cerebrovascular and neurodegenerative changes, contributing to accelerated biological aging, neuroinflammation, and immune dysfunction (179). Although a direct link between FTO and immunosenescence has not yet been established, its association with depression and neuroinflammation places it within the broader context of aging-related immune changes.

Interestingly, while IF has been reported to reduce *FTO* gene expression in overweight and obese individuals, it concurrently enhances BDNF expression via BHB production, a result of metabolic switching (180). This suggests that IF may exert a neuroprotective effect by counteracting the BDNF alterations observed in *FTO* deficiency, potentially supporting neuronal health and cognitive function. Such a possibility underscores the complex, tissue-specific nature of FTO’s functions, in which its downregulation in metabolic tissues may be beneficial. At the same time, its role in the brain remains multifaceted and context-dependent. Further research is warranted to elucidate this relationship and explicitly determine the magnitude of the intercorrelations among *FTO*, BDNF, and IF.

## 7 Evidence supporting the health and immune benefits of intermittent fasting

To our knowledge, no clinical studies have directly tested the effects of IF in frail older adults. Most available research focuses on healthier or middle-aged cohorts, leaving a significant gap in understanding how this nutritional intervention might impact frailty. Given the unique physiological vulnerabilities of frail individuals, targeted research is crucial to assess the safety, feasibility, and potential benefits of fasting regimens in this population. Several human clinical trials have investigated the immunomodulatory and clinical effects of IF in various adult cohorts. These studies provide insights into mechanisms potentially relevant to frailty, such as immune system modulation (82), reduced inflammation (178), and improved metabolic biomarkers when accompanied by a Mediterranean diet (181).

In cancer patients undergoing standard antitumor treatment, repeated cycles of a five-day FMD regimen, consisting of plant-based, calorie-restricted (up to 600 Kcal on day 1; up to 300 Kcal on days 2–5), low-carbohydrate, low protein diet, followed by refeeding led to significant reductions in total and immunosuppressive monocytes, along with increases in activated CD8 + T cells and cytolytic natural killer (NK) cells. These immune changes were accompanied by decreased plasma glucose, insulin, and IGF-1 levels, indicating metabolic improvements that may support immune resilience (182).

In another study of healthy subjects, 72-h water fasting was shown to enhance autophagy by upregulating autophagy-related pathways and downregulating apoptosis-related gene expression, thereby improving leukocyte viability. Additionally, fasting increased peripheral neutrophil counts and enhanced neutrophil degranulation and cytokine secretion (183). Suggesting improved innate immune function through cellular maintenance pathways.

A 30-day single-arm clinical trial investigated the effects of an 8-h time-restricted eating (TRE) window (9 a.m. to 5 p.m.) on markers of immune aging and gut microbiome composition in 49 adults. Participants received nutritionally balanced meals and adhered to the TRE schedule, with no calorie restriction. The study found that TRE significantly reduced markers of immunosenescence, including the percentage of CD4 + CD27^−^CD28^−^ T cells (a senescent T-cell phenotype), indicating a reversal of immunosenescence. Simultaneously, there was a significant increase in the frequency of Th1 cells, which are crucial for antiviral and antitumor immunity, as well as regulatory T cells (Tregs), Th2 cells, T follicular helper (Tfh)-like cells, and B cells. These changes suggest an enhanced and rejuvenated immune response. In contrast, pro-inflammatory Th17 cells declined significantly throughout the intervention, indicating a shift toward a more balanced, less inflammatory immune profile. Furthermore, TRE enhanced T-cell receptor (TCR) diversity and modulated B-cell receptor (BCR) profiles, suggesting a broader, more versatile immune repertoire. Metabolomic analysis revealed increased levels of anti-inflammatory serum metabolites, including sphingosine-1-phosphate, which may contribute to systemic immune regulation. Parallel shifts in the gut microbiome were observed. At baseline (day 0), the microbial environment was dominated by *Firmicutes* (86.37%), with lower proportions of *Bacteroidetes*, *Actinobacteria*, and *Proteobacteria*. After 30 days of TRE, Firmicutes decreased to 7.28%, while *Actinobacteria* and *Bacteroidetes* increased to 16.92 and 3.46%, respectively. Notably, there was a significant increase in the relative abundance of beneficial microbial taxa, such as *Akkermansia* and *Rikenellaceae*, which are often associated with metabolic health, mucosal integrity, and youthful gut profiles in adults following TRE for 30 consecutive days without any dietary restrictions besides IF (184). Collectively, these findings suggest that TRE not only slows immune aging but may actively promote gut microbiota health.

Evidence to date highlights IF as a promising non-pharmacological intervention to improve immune function and systemic health, with potential implications for mitigating frailty and enhancing resilience in aging populations. Table 3 provides a concise overview of human clinical studies evaluating the effects of various IF regimens on immune, metabolic, and clinical outcomes, as well as potential adverse effects. These studies, summarized throughout this review, highlight key findings across different populations and fasting protocols.

**Table 3:** Summary of human clinical studies on intermittent fasting: immune, metabolic, and clinical outcomes.

Study	Population	Type of IF/Fasting regimen	Main outcomes	Potential adverse effects (AEs)
Ezzati et al. (193)	Overweight, older, sedentary adults	TRE (~8 h daytime eating window, ad libitum dietary intake)	Modest, non-significant reductions in IL-1β and TNF-α.	No adverse effects reported.
Malhab et al. (98)	Adults with overweight/obesity	RIF (Dawn-to-dusk fasting)	Upregulation of autophagy genes LAMP2, LC3B, and ATG5; decreased body weight, BMI, fat mass, body fat percent, hip and waist circumferences, LDL, IL-6, and TNF-α; increased HDL, IL-10, and CD163	No adverse effects reported.
Chen et al. (184)	Healthy Chinese adults: subgroups <30 and ≥30 years.	30-day 16:8 TRE (all meals consumed between 09:00–17:00, three canteen-provided meals, energy slightly above estimated daily requirements); control group matched calories but unrestricted eating	Reduced frequency of senescent CD4^+^ T cells (CD4^+^CD27^−^CD28^−^) during TRE (Days 14 and 30), reductions maintained 90 days post-TRE, more pronounced in the ≥30 y group.	No adverse effects reported.
Madkour et al. (70)	Metabolically healthy adults with overweight/obesity	RIF (dawn-to-dusk fasting, ad libitum night-time intake, habitual diet)	Decreased LDL, TG, and DG; increased HDL. Reduced pro-inflammatory cytokines IL-6 and TNF-α, increased anti-inflammatory cytokine IL-10; reductions in plasma sphingosine, sphinganine, sphingosine-1-phosphate, and sphinganine-1-phosphate	No adverse effects reported.
Vernieri et al. (182)	Adults with cancer receiving standard anticancer treatments	FMD (a 5-day plant-based, low-calorie, 600 Kcal on day 1, 300 Kcal/day on days 2–5; low-protein, low-carbohydrate diet)	Downregulation of immunosuppressive myeloid cells (e.g., CD14^+^HLA-DR^−^, CD14^+^PD-L1^+^, CD15^+^) and an increase in activated/cytotoxic T cells (CD8^+^PD-1^+^CD69^+^) and NK cells (CD3^−^CD16^+^CD56dim). Favorable changes in IGF-1 and ketone bodies.	Most common AE: fatigue; other effects included hypoglycemia, syncope, nausea, dizziness, and elevated AST levels. Serious AEs occurred in 4 patients, two attributable to FMD (syncope, severe fatigue). Careful monitoring is recommended for vulnerable patients.
Boujelbane et al. (81)	Sedentary vs. physically active older adults.	Ramadan diurnal IF (dawn-to-dusk fasting)	Physically active group: significant improvement in executive function, attention, inhibition, associative memory, and recognition memory; sedentary group: considerable reduction in associative learning performance	Poor sleep quality and excessive daytime sleepiness were significantly higher in the sedentary group.
Qian et al. (183)	Healthy adults (aged 26–60)	Short-term intensive fasting (72-h water-only fast under supervision)	Enhanced innate immune function, by upregulation of autophagy machinery in leukocytes, reduced apoptosis levels, and increased neutrophil counts and activation	No adverse effects reported.
Currenti et al. (189)	Italian adults	Self-selected time-restricted eating (participants followed TRF-8 or TRF-10 schedules, Mediterranean diet; cross-sectional observational study; no prescribed caloric restriction)	TRF-10 was inversely associated with overweight/obesity, hypertension, and dyslipidemias, TRF-8 was inversely associated with overweight/obesity and hypertension. No associations were found with type 2 diabetes.	No adverse effects reported.
Ooi et al. (76)	Malaysian older adults ≥ 60 years, with mild cognitive impairment	“Islamic sunnah” IF (non-consecutive Monday and Thursdays)	Better cognitive performance, Higher “successful aging” rates, increased superoxide dismutase (SOD) activity, and reduced body weight, insulin, fasting blood glucose, malondialdehyde (MDA), C-reactive protein (CRP), and DNA damage	No adverse effects reported.
Anton et al. (77)	Overweight/obese sedentary older adults (>65 years), with mild to moderate functional limitations.	Daily TRE (~8 h eating window, ad libitum)	Significant but modest weight loss; no significant changes in cognitive or physical functions; small, non-significant improvements in mental and physical quality of life	Few adverse effects: Two participants reported headaches during fasting (resolved with hydration), one reported dizziness (determined after a small snack)
Jamshed et al. (97)	Overweight/obese healthy adults	Early TRE (6-h eating window, isocaloric to habitual intake)	Significant improvement in 24-h glucose profile, insulin sensitivity; significant increases in expression of several circadian clock genes (BMAL1, CRY1/2, RORA), SIRT1, and autophagy gene LC3A; most other genes unchanged.	One participant (who later withdrew) experienced nausea and vomiting while following the control schedule.
Madkour et al. (86)	Overweight/obese healthy adults	RIF (dawn-to-dusk fasting, ad libitum night-time intake; no imposed caloric restriction)	Upregulation of SOD2, TFAM, NRF2, SIRT1/SIRT3; improved oxidative stress profile	No adverse effects reported.
Trepanowski (190)	Metabolically healthy obese adults	ADF (24 h fast, ~25% of energy needs, alternating with 24 h ad libitum vs. CR group	Similar weight loss and cardiometabolic improvements to daily CR; greater loss of lean mass in ADF; no superiority in weight loss, maintenance, or cardiovascular risk markers	No adverse effects reported.
Faris et al. (87)	Healthy adults	RIF (dawn-to-dusk fasting with usual night-time meals)	Significant reduction in pro-inflammatory cytokines: IL-6, IL-1β, and TNF-α.	No adverse effects reported.

RIF, Ramadan intermittent fasting; IL-1β, interleukin-1 beta; IL-6, interleukin-6; LAMP2, lysosome-associated membrane protein 2; LC3B, microtubule-associated protein 1 light chain 3 beta; ATG5, autophagy-related gene 5; TNF-α, tumor necrosis factor alpha; CD163, cluster of differentiation 163; DG, diacylglycerols; FMD, fasting-mimicking diet; NK, natural killer (cells); BMAL1, brain and muscle ARNT-like protein 1; CRY1/2, cryptochrome 1 and 2; RORA, retinoic acid receptor-related orphan receptor alpha; SIRT1, sirtuin 1; SOD2, superoxide dismutase 2; TFAM, mitochondrial transcription factor A; NRF2, nuclear factor erythroid 2–related factor 2; ADF, alternate-day fasting.

## 8 Population diversity, generalizability, and safety considerations

While evidence supporting the health and immune benefits of IF continues to grow, it is essential to acknowledge the limited diversity of populations in existing studies. Most clinical trials on IF have predominantly enrolled healthy, middle-aged adults, often excluding older individuals with comorbidities, sarcopenia, or polypharmacy, features that commonly accompany frailty. This limits the generalizability of findings to older adults, particularly those most at risk of immunosenescence, inflammaging, and functional decline (4, 185).

Older adults exhibit distinct physiological characteristics that may sharpen their response to IF, including altered circadian rhythms, reduced metabolic flexibility, loss of muscle and bone mass, and impaired immune function. Furthermore, elderly individuals are more susceptible to adverse events such as hypoglycemia, dehydration, headaches, fatigue, and nutrient deficiencies, particularly if calorie intake and nutrient density are not carefully maintained during feeding windows (4, 186). Evidence from a recent review of IF effects in middle-aged and older adults indicates that TRE and 5:2 regimens were generally well tolerated, with relatively few adverse events reported in short-term studies. However, increased risk of hypoglycemia was identified in older adults with type 2 diabetes, underscoring the need for caution in metabolically vulnerable individuals. Moreover, because the available studies were small and short in duration and mostly limited to healthy participants, potential risks, sarcopenia, and functional decline may be underrecognized (187). Notably, a recent meta-analysis including IF studies in middle-aged, overweight/obese adults found that IF was not associated with significantly increased risk of common adverse effects, compared with control diets. Serious adverse events were rare and not attributed to IF interventions (188). However, these findings may not be generalized to the target population of our review. Accordingly, extrapolation to elderly individuals, especially those with comorbidities, sarcopenia, or polypharmacy, remains uncertain, reinforcing the need for targeted research in that demographic.

Additional sources of variability in this demographic include sex differences, cognitive status, baseline nutritional adequacy, and physical activity levels, all of which complicate the design and evaluation of IF interventions. While a few human trials have suggested that IF may enhance immune resilience and reduce inflammaging, these effects cannot yet be confidently extrapolated to frail older adults without targeted investigation (33, 186).

## 9 Conclusion

IF has emerged as a promising nutritional strategy to modulate immune function, reduce systemic inflammation, and support metabolic health, all of which are key factors in immunosenescence and frailty. Preclinical and clinical studies have demonstrated favorable shifts in immune cell phenotypes, reduction in markers of immune aging, enhanced gut microbiota diversity, and improvements in cardiometabolic risk factors. These findings highlight IF as a potential non-pharmacological approach to strengthen immune resilience and promote healthy aging.

However, evidence in frail and older populations remains limited. To establish safety and efficacy, further clinical trials should stratify participants by age, frailty status, and sex. Outcomes relevant to older adults should be included, such as physical functions, quality of life, immune biomarkers, and infection risk. Such data will be crucial to determine whether IF can be implemented as a safe intervention to enhance resilience and delay frailty progression in the aging population.

### 9.1 Gaps and future research directions

Despite growing evidence linking intermittent fasting and immunonutrition to reduced inflammation and improved immune function during aging, significant knowledge gaps persist. Future research should prioritize large, well-designed randomized controlled trials in diverse elderly populations to confirm long-term efficacy and safety. Mechanistic studies are needed to elucidate cellular and molecular pathways modulated by fasting regimens, particularly those related to immune cell metabolism and neuroinflammation. Standardizing definitions of fasting and the measurement of immune and cognitive outcomes will enable synthesis across studies. Additionally, research on personalized fasting interventions that account for frailty, comorbidities, and nutritional status is essential to optimize benefits for healthy aging.

### 9.2 Limitations

This narrative review has inherent limitations due to its flexible and non-systematic approach. The selection of studies was based on the author’s discretion, which may introduce selection bias and limit comprehensiveness. The lack of a standardized search and inclusion protocol reduces reproducibility and transparency. Important studies might have been overlooked, and the interpretation of evidence depends heavily on the authors’ perspective. Therefore, while this review provides a broad overview and conceptual insights, definitive conclusions and clinical recommendations should be made cautiously.

## Glossary

AcAc: Acetoacetate
ADF: Alternate-Day Fasting
AMPK: AMP-Activated Protein Kinase
ATF4: Activating Transcription Factor 4
BCR: B-cell Receptor
BDNF: Brain-Derived Neurotrophic Factor
BHB: Beta-Hydroxybutyrate
CD: Cluster of Differentiation
CMS: Cyclic Metabolic Switching
CNS: Central Nervous System
CR: Calorie Restriction
CRP: C-Reactive Protein
CTSS: Cathepsin S
DAMPs: Damage-Associated Molecular Patterns
FFA: Free Fatty Acids
FGF21: Fibroblast Growth Factor 21
FMD: Fasting-Mimicking Diet
FMO3: Flavin-containing Monooxygenase 3
FOXO: Forkhead Box O (transcription factors)
GFAP: Glial Fibrillary Acidic Protein
GLP-1: Glucagon-Like Peptide 1
GWAS: Genome-Wide Association Study
HDL: High-Density Lipoprotein
HDACs: Histone Deacetylases
hs-CRP: High-Sensitivity C-reactive Protein
IF: Intermittent Fasting
IGF-1: Insulin-Like Growth Factor 1
IL: Interleukin
JAK2: Janus Kinase 2
LDL: Low-Density Lipoprotein
MCT: Monocarboxylate Transporter
MCTs: Monocarboxylate Transporters
MHC II: Major Histocompatibility Complex II
mTOR: Mammalian Target of Rapamycin
mtDNA: Mitochondrial DNA
mtRNA: Mitochondrial RNA
NAD: Nicotinamide Adenine Dinucleotide
NK: Natural Killer (cell)
NLRP3: NOD-like Receptor Family Pyrin Domain Containing 3
PDK4: Pyruvate Dehydrogenase Kinase 4
PF: Periodic Fasting
PKA: Protein Kinase A
PPAR-: Peroxisome Proliferator-Activated Receptor Alpha
RIF: Ramadan Intermittent Fasting
ROS: Reactive Oxygen Species
SASP: Senescence-Associated Secretory Phenotype
SCFAs: Short-chain Fatty Acids
SIRT/SIRT1: Sirtuins/Sirtuin 1
SNP: Single Nucleotide Polymorphism
STAT3: Signal Transducer and Activator of Transcription 3
TCA: Tricarboxylic Acid
TCR: T-cell Receptor
Tfh: T Follicular Helper Cell
Th: T Helper Cell
TLRs: Toll-Like Receptors
TMAO: Trimethylamine N-oxide
TNF-α: Tumor Necrosis Factor-alpha
TRE: Time-Restricted Eating
Treg: Regulatory T Cell
VLDL: Very Low-Density Lipoprotein
ZO-1: Zonula Occludens 1

## References

[1] WHO. Ageing and health. 2024. Available online at: https://www.who.int/news-room/fact-sheets/detail/ageing-and-health (Accessed June 5, 2025).

[2] Clegg A . Frailty in elderly people. Lancet. (2013) 381:752–62. 23395245 10.1016/S0140-6736(12)62167-9 PMC4098658

[3] Rose MR Flatt T Graves JL Greer LF Martinez DE Matos M . What is aging? Front Genet. (2012) 3:134. doi: 10.3389/fgene.2012.00134, 22833755 PMC3400891

[4] López-Otín C Blasco MA Partridge L Serrano M Kroemer G. The hallmarks of aging. Cell. (2013) 153:1194–217. doi: 10.1016/j.cell.2013.05.039, 23746838 PMC3836174

[5] WHO. WHO clinical consortium on healthy ageing: topic focus: frailty and intrinsic capacity: report of consortium meeting, 1–2 December 2016 in Geneva, Switzerland. in WHO clinical consortium on healthy ageing: topic focus: frailty and intrinsic capacity: report of consortium meeting, 1–2 December 2016 in Geneva, Switzerland 2017

[6] Sepúlveda M Arauna D García F Albala C Palomo I Fuentes E. Frailty in aging and the search for the optimal biomarker: a review. Biomedicine. (2022) 10:1426. doi: 10.3390/biomedicines10061426, 35740447 PMC9219911

[7] Liu Z Liang Q Ren Y Guo C Ge X Wang L . Immunosenescence: molecular mechanisms and diseases. Signal Transduct Target Ther. (2023) 8:200. doi: 10.1038/s41392-023-01451-2, 37179335 PMC10182360

[8] Dugan B Conway J Duggal NA. Inflammaging as a target for healthy ageing. Age Ageing. (2023) 52:328. doi: 10.1093/ageing/afac328 36735849

[9] Furman D Campisi J Verdin E Carrera-Bastos P Targ S Franceschi C . Chronic inflammation in the etiology of disease across the life span. Nat Med. (2019) 25:1822–32. doi: 10.1038/s41591-019-0675-0, 31806905 PMC7147972

[10] Claro-Cala CM Rivero-Pino F Torrecillas-López M Jimenez-Gonzalez V Montserrat-de la Paz S. Immunonutrition: future perspective in neurodegenerative disorders. Nutr Neurosci. (2025) 28:807–18. doi: 10.1080/1028415X.2024.2425565, 39561029

[11] Meydani SN das S Pieper CF Lewis MR Klein S Dixit VD . Long-term moderate calorie restriction inhibits inflammation without impairing cell-mediated immunity: a randomized controlled trial in non-obese humans. Aging (Albany NY). (2016) 8:1416–31. doi: 10.18632/aging.100994, 27410480 PMC4993339

[12] Tizazu AM. Fasting and calorie restriction modulate age-associated immunosenescence and inflammaging. Aging Medicine. (2024) 7:499–509. doi: 10.1002/agm2.12342, 39234195 PMC11369340

[13] Sun M-L Yao W Wang XY Gao S Varady KA Forslund SK . Intermittent fasting and health outcomes: an umbrella review of systematic reviews and meta-analyses of randomised controlled trials. EClinicalMedicine. (2024) 70:102519. doi: 10.1016/j.eclinm.2024.102519, 38500840 PMC10945168

[14] Koppold DA Breinlinger C Hanslian E Kessler C Cramer H Khokhar AR . International consensus on fasting terminology. Cell Metab. (2024) 36:1779–1794.e4. doi: 10.1016/j.cmet.2024.06.013, 39059384 PMC11504329

[15] Gudden J Arias Vasquez A Bloemendaal M. The effects of intermittent fasting on brain and cognitive function. Nutrients. (2021) 13:3166. doi: 10.3390/nu13093166, 34579042 PMC8470960

[16] Patterson RE Sears DD. Metabolic effects of intermittent fasting. Annu Rev Nutr. (2017) 37:371–93. 28715993 10.1146/annurev-nutr-071816-064634 PMC13170603

[17] Semnani-Azad Z . Intermittent fasting strategies and their effects on body weight and other cardiometabolic risk factors: systematic review and network meta-analysis of randomised clinical trials. BMJ. (2025):389. 10.1136/bmj-2024-082007 PMC12175170 40533200

[18] Zhang H Puleston DJ Simon AK. Autophagy and immune senescence. Trends Mol Med. (2016) 22:671–86. doi: 10.1016/j.molmed.2016.06.001, 27395769

[19] Teissier T Boulanger E Cox LS. Interconnections between inflammageing and immunosenescence during ageing. Cells. (2022) 11:359. doi: 10.3390/cells11030359, 35159168 PMC8834134

[20] Müller L Di Benedetto S. Inflammaging, immunosenescence, and cardiovascular aging: insights into long COVID implications. Frontiers in Cardiovascular Medicine. (2024) 11:1384996. 38988667 10.3389/fcvm.2024.1384996 PMC11233824

[21] Hernandez-Segura A Nehme J Demaria M. Hallmarks of cellular senescence. Trends Cell Biol. (2018) 28:436–53. doi: 10.1016/j.tcb.2018.02.001, 29477613

[22] Kawai T Akira S. Toll-like receptors and their crosstalk with other innate receptors in infection and immunity. Immunity. (2011) 34:637–50. doi: 10.1016/j.immuni.2011.05.006, 21616434

[23] Netea MG van der Meer JW. Trained immunity: an ancient way of remembering. Cell Host Microbe. (2017) 21:297–300. doi: 10.1016/j.chom.2017.02.003, 28279335

[24] Xu X Pang Y Fan X. Mitochondria in oxidative stress, inflammation and aging: from mechanisms to therapeutic advances. Signal Transduct Target Ther. (2025) 10:190. doi: 10.1038/s41392-025-02253-4, 40500258 PMC12159213

[25] Caldarelli M Rio P Marrone A Giambra V Gasbarrini A Gambassi G . Inflammaging: the next challenge—exploring the role of gut microbiota, environmental factors, and sex differences. Biomedicine. (2024) 12:1716. doi: 10.3390/biomedicines12081716, 39200181 PMC11351301

[26] Biagi E Nylund L Candela M Ostan R Bucci L Pini E . Through ageing, and beyond: gut microbiota and inflammatory status in seniors and centenarians. PLoS One. (2010) 5:e10667. doi: 10.1371/journal.pone.0010667, 20498852 PMC2871786

[27] Khaledi M Poureslamfar B Alsaab HO Tafaghodi S Hjazi A Singh R . The role of gut microbiota in human metabolism and inflammatory diseases: a focus on elderly individuals. Ann Microbiol. (2024) 74:1. doi: 10.1186/s13213-023-01744-5

[28] Ren J Li H Zeng G Pang B Wang Q Wei J. Gut microbiome-mediated mechanisms in aging-related diseases: are probiotics ready for prime time? Front Pharmacol. (2023) 14:1178596. doi: 10.3389/fphar.2023.1178596, 37324466 PMC10267478

[29] Pangrazzi L Meryk A. Molecular and cellular mechanisms of Immunosenescence: modulation through interventions and lifestyle changes. Biology. (2024) 14:17. doi: 10.3390/biology14010017, 39857248 PMC11760833

[30] O’Neill LA Pearce EJ. Immunometabolism governs dendritic cell and macrophage function. J Exp Med. (2016) 213:15–23. doi: 10.1084/jem.20151570, 26694970 PMC4710204

[31] Li X Li C Zhang W Wang Y Qian P Huang H. Inflammation and aging: signaling pathways and intervention therapies. Signal Transduct Target Ther. (2023) 8:239. doi: 10.1038/s41392-023-01502-8, 37291105 PMC10248351

[32] Nikolich-Žugich J. The twilight of immunity: emerging concepts in aging of the immune system. Nat Immunol. (2018) 19:10–9. doi: 10.1038/s41590-017-0006-x, 29242543

[33] Mattson MP . Intermittent metabolic switching, neuroplasticity and brain health. Nat Rev Neurosci. (2018) 19:81–94. 10.1038/nrn.2017.156 PMC5913738 29321682

[34] Youm Y-H Nguyen KY Grant RW Goldberg EL Bodogai M Kim D . The ketone metabolite β-hydroxybutyrate blocks NLRP3 inflammasome–mediated inflammatory disease. Nat Med. (2015) 21:263–9. doi: 10.1038/nm.3804, 25686106 PMC4352123

[35] Affourtit C Carré JE. Mitochondrial involvement in sarcopenia. Acta Physiol. (2024) 240:e14107. doi: 10.1111/apha.14107 38304924

[36] Valenzuela-Ahumada LA . Fasting the mitochondria to prevent neurodegeneration: the role of ceramides. Front Neurosci. (2025) 19:1602149. 40535970 10.3389/fnins.2025.1602149 PMC12174400

[37] Hein ZM Arbain MFF Kumar S Mehat MZ Hamid HA Che Ramli MD . Intermittent fasting as a neuroprotective strategy: gut-brain Axis modulation and metabolic reprogramming in neurodegenerative disorders. Nutrients. (2025) 17. doi: 10.3390/nu17142266, 40732891 PMC12298811

[38] Cheng C-W Adams GB Perin L Wei M Zhou X Lam BS . Prolonged fasting reduces IGF-1/PKA to promote hematopoietic-stem-cell-based regeneration and reverse immunosuppression. Cell Stem Cell. (2014) 14:810–23. doi: 10.1016/j.stem.2014.04.014, 24905167 PMC4102383

[39] James DL Hawley NA Mohr AE Hermer J Ofori E Yu F . Impact of intermittent fasting and/or caloric restriction on aging-related outcomes in adults: a scoping review of randomized controlled trials. Nutrients. (2024) 16:316. doi: 10.3390/nu16020316, 38276554 PMC10820472

[40] Templeman I Smith HA Chowdhury E Chen YC Carroll H Johnson-Bonson D . A randomized controlled trial to isolate the effects of fasting and energy restriction on weight loss and metabolic health in lean adults. Sci Transl Med. (2021) 13:8034. doi: 10.1126/scitranslmed.abd8034, 34135111

[41] Lowe DA Wu N Rohdin-Bibby L Moore AH Kelly N Liu YE . Effects of time-restricted eating on weight loss and other metabolic parameters in women and men with overweight and obesity: the TREAT randomized clinical trial. JAMA Intern Med. (2020) 180:1491–9. doi: 10.1001/jamainternmed.2020.4153, 32986097 PMC7522780

[42] Howlett SE Rutenberg AD Rockwood K. The degree of frailty as a translational measure of health in aging. Nature Aging. (2021) 1:651–65. doi: 10.1038/s43587-021-00099-3, 37117769

[43] Fried LP Cohen AA Xue QL Walston J Bandeen-Roche K Varadhan R. The physical frailty syndrome as a transition from homeostatic symphony to cacophony. Nature Aging. (2021) 1:36–46. doi: 10.1038/s43587-020-00017-z, 34476409 PMC8409463

[44] Kim DH Rockwood K. Frailty in older adults. N Engl J Med. (2024) 391:538–48. doi: 10.1056/NEJMra2301292, 39115063 PMC11634188

[45] O’Caoimh R . Prevalence of frailty in 62 countries across the world: a systematic review and meta-analysis of population-level studies. Age Ageing. (2021) 50:96–104. doi: 10.1093/ageing/afaa219, 33068107

[46] Fried LP . Untangling the concepts of disability, frailty, and comorbidity: implications for improved targeting and care. J Gerontol Ser A Biol Med Sci. (2004) 59:M255–63. 10.1093/gerona/59.3.m255 15031310

[47] Buondonno I Sassi F Cattaneo F D’Amelio P. Association between immunosenescence, mitochondrial dysfunction and frailty syndrome in older adults. Cells. (2022) 12:44. doi: 10.3390/cells12010044, 36611837 PMC9818926

[48] Pera A Campos C López N Hassouneh F Alonso C Tarazona R . Immunosenescence: implications for response to infection and vaccination in older people. Maturitas. (2015) 82:50–5. doi: 10.1016/j.maturitas.2015.05.004, 26044074

[49] Fulop T Larbi A Dupuis G Le Page A Frost EH Cohen AA . Immunosenescence and inflamm-aging as two sides of the same coin: friends or foes? Front Immunol. (2018) 8:1960. doi: 10.3389/fimmu.2017.01960, 29375577 PMC5767595

[50] Lee GC Restrepo MI Harper N Manoharan MS Smith AM Meunier JA . Immunologic resilience and COVID-19 survival advantage. J Allergy Clin Immunol. (2021) 148:1176–91. doi: 10.1016/j.jaci.2021.08.021, 34508765 PMC8425719

[51] Yao X Li H Leng SX. Inflammation and immune system alterations in frailty. Clin Geriatr Med. (2011) 27:79–87. doi: 10.1016/j.cger.2010.08.002, 21093724 PMC3011971

[52] De Cabo R Mattson MP. Effects of intermittent fasting on health, aging, and disease. N Engl J Med. (2019) 381:2541–51. doi: 10.1056/NEJMra1905136, 31881139

[53] Teleanu DM Niculescu AG Lungu II Radu CI Vladâcenco O Roza E . An overview of oxidative stress, neuroinflammation, and neurodegenerative diseases. Int J Mol Sci. (2022) 23:5938. doi: 10.3390/ijms23115938, 35682615 PMC9180653

[54] Erickson MA Banks WA. Age-associated changes in the immune system and blood–brain barrier functions. Int J Mol Sci. (2019) 20:1632. doi: 10.3390/ijms20071632, 30986918 PMC6479894

[55] Norden DM Godbout J. Microglia of the aged brain: primed to be activated and resistant to regulation. Neuropathol Appl Neurobiol. (2013) 39:19–34. doi: 10.1111/j.1365-2990.2012.01306.x, 23039106 PMC3553257

[56] Perry VH Teeling J. Microglia and macrophages of the central nervous system: the contribution of microglia priming and systemic inflammation to chronic neurodegeneration. Seminars in immunopathology, (2013) 35:601–612. Springer. 23732506 10.1007/s00281-013-0382-8 PMC3742955

[57] Rajput M Malik IA Methi A Cortés Silva JA Fey D Wirths O . Cognitive decline and neuroinflammation in a mouse model of obesity: an accelerating role of ageing. Brain Behav Immun. (2025) 125:226–39. doi: 10.1016/j.bbi.2024.12.154, 39730092

[58] Thadathil N Nicklas EH Mohammed S Lewis TL Jr Richardson A Deepa SS. Necroptosis increases with age in the brain and contributes to age-related neuroinflammation. Geroscience. (2021) 43:2345–61. doi: 10.1007/s11357-021-00448-5, 34515928 PMC8599532

[59] Liu PP Liu XH Ren MJ Liu XT Shi XQ Li ML . Neuronal cathepsin S increases neuroinflammation and causes cognitive decline via CX3CL1-CX3CR1 axis and JAK2-STAT3 pathway in aging and Alzheimer's disease. Aging Cell. (2025) 24:e14393. doi: 10.1111/acel.14393, 39453382 PMC11822647

[60] Jurgens HA Johnson RW. Dysregulated neuronal–microglial cross-talk during aging, stress and inflammation. Exp Neurol. (2012) 233:40–8. doi: 10.1016/j.expneurol.2010.11.014, 21110971 PMC3071456

[61] Liang R Qi X Cai Q Niu L Huang X Zhang D . The role of NLRP3 inflammasome in aging and age-related diseases. Immun Ageing. (2024) 21:14. doi: 10.1186/s12979-023-00395-z, 38317229 PMC10840156

[62] Mottahedin A . Effect of neuroinflammation on synaptic organization and function in the developing brain: implications for neurodevelopmental and neurodegenerative disorders. Front Cell Neurosci. (2017) 11:190. 28744200 10.3389/fncel.2017.00190 PMC5504097

[63] Alkurd R Mahrous L Zeb F Khan MAB Alhaj H Khraiwesh HM . Effect of calorie restriction and intermittent fasting regimens on brain-derived neurotrophic factor levels and cognitive function in humans: a systematic review. Medicina. (2024) 60:191. doi: 10.3390/medicina60010191, 38276070 PMC10819730

[64] Mattson MP. The cyclic metabolic switching theory of intermittent fasting. Nat Metab. (2025) 7:665–78. doi: 10.1038/s42255-025-01254-5, 40087409

[65] Soliman S . Switching host metabolism as an approach to dampen SARS-CoV-2 infection. Ann Nutr Metab. (2021) 76:297–303. 10.1159/000510508 PMC7573915 32950986

[66] Elhag MR . Transforming gut health through Ramadan intermittent fasting: a review on metabolic and Microbiomic insights. Clinical Nutrition ESPEN. (2025) 69:115–30. doi: 10.1016/j.clnesp.2025.06.051, 40609897

[67] Khan MN Khan SI Rana MI Ayyaz A Khan MY Imran M. Intermittent fasting positively modulates human gut microbial diversity and ameliorates blood lipid profile. Front Microbiol. (2022) 13. doi: 10.3389/fmicb.2022.922727, 36081793 PMC9445987

[68] Zeb F Osaili T Obaid R Naja F Radwan H Cheikh Ismail L . Gut microbiota and time-restricted feeding/eating: a targeted biomarker and approach in precision nutrition. Nutrients. (2023) 15. doi: 10.3390/nu15020259, 36678130 PMC9863108

[69] Wang W Dernst A Martin B Lorenzi L Cadefau-Fabregat M Phulphagar K . Butyrate and propionate are microbial danger signals that activate the NLRP3 inflammasome in human macrophages upon TLR stimulation. Cell Rep. (2024) 43:114736. doi: 10.1016/j.celrep.2024.114736, 39277863

[70] Madkour MI Islam MT Tippetts TS Chowdhury KH Lesniewski LA Summers SA . Ramadan intermittent fasting is associated with ameliorated inflammatory markers and improved plasma sphingolipids/ceramides in subjects with obesity: lipidomics analysis. Sci Rep. (2023) 13:17322. doi: 10.1038/s41598-023-43862-9, 37833312 PMC10576029

[71] Lu L . The effect of intermittent fasting on insulin resistance, lipid profile, and inflammation on metabolic syndrome: a GRADE assessed systematic review and meta-analysis. J Health Popul Nutr. (2025) 44:025–01039. 10.1186/s41043-025-01039-2 PMC12363089 40826125

[72] Nomura M . A ketogenic diet reduces age-induced chronic neuroinflammation in mice running title: ketogenic diet and brain inflammaging. bioRxiv. (2023)

[73] Wang W Cao W Zhang S Chen D Liu L. The role of calprotectin in the diagnosis and treatment of inflammatory bowel disease. Int J Mol Sci. (2025) 26:1996. doi: 10.3390/ijms26051996, 40076618 PMC11900593

[74] Sharifi S Rostami F Babaei Khorzoughi K Rahmati M. Effect of time-restricted eating and intermittent fasting on cognitive function and mental health in older adults: a systematic review. Prev Med Rep. (2024) 42:102757. doi: 10.1016/j.pmedr.2024.102757, 38774517 PMC11107340

[75] James D Sears D Larkey L Maxfield M Ofori E Han SY . *Prolonged nightly fasting among older adults: a pilot study exploring changes in cognitive function.* Innovation. Aging. (2022) 6:823–3. doi: 10.1093/geroni/igac059.2960 PMC11736298 39830610

[76] Ooi TC Meramat A Rajab NF Shahar S Ismail IS Azam AA . Intermittent fasting enhanced the cognitive function in older adults with mild cognitive impairment by inducing biochemical and metabolic changes: a 3-year progressive study. Nutrients. (2020) 12:2644. doi: 10.3390/nu12092644, 32872655 PMC7551340

[77] Anton SD Lee SA Donahoo WT McLaren C Manini T Leeuwenburgh C . The effects of time restricted feeding on overweight, older adults: a pilot study. Nutrients. (2019) 11:1500. doi: 10.3390/nu11071500, 31262054 PMC6682944

[78] Currenti W Godos J Castellano S Caruso G Ferri R Caraci F . Time-restricted feeding is associated with mental health in elderly Italian adults. Chronobiol Int. (2021) 38:1507–16. doi: 10.1080/07420528.2021.1932998, 34100325

[79] Currenti W Godos J Castellano S Caruso G Ferri R Caraci F . Association between time restricted feeding and cognitive status in older Italian adults. Nutrients. (2021) 13:191. doi: 10.3390/nu13010191, 33435416 PMC7827225

[80] Ooi T . Antioxidant potential, DNA damage, inflammation, glycemic control and lipid metabolism alteration: a mediation analysis of islamic sunnah intermittent fasting on cognitive function among older adults with mild cognitive impairment. J Nutrition Health Aging. (2022) 26:272–81. 35297471 10.1007/s12603-022-1757-0 PMC12275610

[81] Boujelbane MA . Time-restricted feeding and cognitive function in sedentary and physically active elderly individuals: Ramadan diurnal intermittent fasting as a model. Front Nutr. (2022) 9:1041216. 36438750 10.3389/fnut.2022.1041216 PMC9682201

[82] Gasmi M Silvia Hardiany N van der Merwe M Martins IJ Sharma A Williams-Hooker R. The influence of time-restricted eating/feeding on Alzheimer’s biomarkers and gut microbiota. Nutr Neurosci. (2025) 28:156–70. doi: 10.1080/1028415X.2024.2359868, 38953237

[83] Espeland MA Luchsinger JA Baker LD Neiberg R Kahn SE Arnold SE . Effect of a long-term intensive lifestyle intervention on prevalence of cognitive impairment. Neurology. (2017) 88:2026–35. doi: 10.1212/WNL.0000000000003955, 28446656 PMC5440245

[84] Carmichael OT Neiberg RH Dutton GR Hayden KM Horton E Pi-Sunyer FX . Long-term change in physiological markers and cognitive performance in type 2 diabetes: the look AHEAD study. J Clin Endocrinol Metabol. (2020) 105:e4778–91. doi: 10.1210/clinem/dgaa591, 32845968 PMC7566388

[85] Haasis E Bettenburg A Lorentz A. Effect of intermittent fasting on immune parameters and intestinal inflammation. Nutrients. (2024) 16:3956. doi: 10.3390/nu16223956, 39599741 PMC11597193

[86] Madkour MI . Ramadan diurnal intermittent fasting modulates SOD2, TFAM, Nrf2, and sirtuins (SIRT1, SIRT3) gene expressions in subjects with overweight and obesity. Diabetes Res Clin Pract. (2019) 155:107801 31356832 10.1016/j.diabres.2019.107801

[87] Faris MA Kacimi S Al-Kurd RA Fararjeh MA Bustanji YK Mohammad MK . Intermittent fasting during Ramadan attenuates proinflammatory cytokines and immune cells in healthy subjects. Nutr Res. (2012) 32:947–55. doi: 10.1016/j.nutres.2012.06.021, 23244540

[88] Faris ME Madkour MI Obaideen AK Dalah EZ Hasan HA Radwan H . Effect of Ramadan diurnal fasting on visceral adiposity and serum adipokines in overweight and obese individuals. Diabetes Res Clin Pract. (2019) 153:166–75. 31150725 10.1016/j.diabres.2019.05.023

[89] MAE F . Impact of diurnal intermittent fasting during Ramadan on inflammatory and oxidative stress markers in healthy people: systematic review and meta-analysis. J Nutrition Intermediary Metabolism. (2019) 15:18–26.

[90] Aamir AB Kumari R Latif R Ahmad S Rafique N Salem AM . Effects of intermittent fasting and caloric restriction on inflammatory biomarkers in individuals with obesity/overweight: a systematic review and meta-analysis of randomized controlled trials. Obes Rev. (2025) 26:e13838. doi: 10.1111/obr.13838, 39289905

[91] Khalafi M Habibi Maleki A Mojtahedi S Ehsanifar M Rosenkranz SK Symonds ME . The effects of intermittent fasting on inflammatory markers in adults: a systematic review and pairwise and network Meta-analyses. Nutrients. (2025) 17:2388. doi: 10.3390/nu17152388, 40805975 PMC12348594

[92] de Ciutiis I Djakovic S Cagigas ML Masedunskas A Smith L Franceschi C . Long-term fasting and its influence on inflammatory biomarkers: a comprehensive scoping review. Ageing Res Rev. (2025) 110:102797. doi: 10.1016/j.arr.2025.102797, 40484176

[93] de Toledo FW . Safety, health improvement and well-being during a 4 to 21-day fasting period in an observational study including 1422 subjects. PLoS One. (2019) 14:e0209353. doi: 10.1371/journal.pone.0209353, 30601864 PMC6314618

[94] Liu X Wei J Ma Z He Y. Rapamycin-and starvation-induced autophagy are associated with miRNA dysregulation in A549 cells. Acta Biochim Biophys Sin. (2019) 51:393–401. doi: 10.1093/abbs/gmz022, 30908573

[95] Decuypere J-P van Giel D Janssens P Dong K Somlo S Cai Y . Interdependent regulation of polycystin expression influences starvation-induced autophagy and cell death. Int J Mol Sci. (2021) 22:13511. doi: 10.3390/ijms222413511, 34948309 PMC8706473

[96] Mizushima N Levine B. Autophagy in human diseases. N Engl J Med. (2020) 383:1564–76. doi: 10.1056/NEJMra2022774, 33053285

[97] Jamshed H Beyl R Della Manna D Yang E Ravussin E Peterson C. Early time-restricted feeding improves 24-hour glucose levels and affects markers of the circadian clock, aging, and autophagy in humans. Nutrients. (2019) 11:1234. doi: 10.3390/nu11061234, 31151228 PMC6627766

[98] Malhab LJB . Dawn-to-dusk intermittent fasting is associated with overexpression of autophagy genes: a prospective study on overweight and obese cohort. Clinical Nutrition ESPEN. (2025) 65:209–17. 39542136 10.1016/j.clnesp.2024.11.002

[99] Singh R Kaushik S Wang Y Xiang Y Novak I Komatsu M . Autophagy regulates lipid metabolism. Nature. (2009) 458:1131–5. doi: 10.1038/nature07976, 19339967 PMC2676208

[100] Deretic V Levine B. Autophagy balances inflammation in innate immunity. Autophagy. (2018) 14:243–51. doi: 10.1080/15548627.2017.1402992, 29165043 PMC5902214

[101] Riffelmacher T Richter FC Simon AK. Autophagy dictates metabolism and differentiation of inflammatory immune cells. Autophagy. (2018) 14:199–206. doi: 10.1080/15548627.2017.1362525, 28806133 PMC5902226

[102] Gutierrez MG Master SS Singh SB Taylor GA Colombo MI Deretic V. Autophagy is a defense mechanism inhibiting BCG and Mycobacterium tuberculosis survival in infected macrophages. Cell. (2004) 119:753–66. 15607973 10.1016/j.cell.2004.11.038

[103] Nakagawa I Amano A Mizushima N Yamamoto A Yamaguchi H Kamimoto T . Autophagy defends cells against invading group a Streptococcus. Science. (2004) 306:1037–40. 15528445 10.1126/science.1103966

[104] Deretic V. Autophagy in inflammation, infection, and immunometabolism. Immunity. (2021) 54:437–53. doi: 10.1016/j.immuni.2021.01.018, 33691134 PMC8026106

[105] Hatori M Vollmers C Zarrinpar A DiTacchio L Bushong EA Gill S . Time-restricted feeding without reducing caloric intake prevents metabolic diseases in mice fed a high-fat diet. Cell Metab. (2012) 15:848–60. doi: 10.1016/j.cmet.2012.04.019, 22608008 PMC3491655

[106] Bujak AL Crane JD Lally JS Ford RJ Kang SJ Rebalka IA . AMPK activation of muscle autophagy prevents fasting-induced hypoglycemia and myopathy during aging. Cell Metab. (2015) 21:883–90. doi: 10.1016/j.cmet.2015.05.016, 26039451 PMC5233441

[107] Iglesias M Felix DA Gutiérrez-Gutiérrez Ó de Miguel-Bonet MDM Sahu S Fernández-Varas B . Downregulation of mTOR signaling increases stem cell population telomere length during starvation of immortal planarians. Stem cell Reports. (2019) 13:405–18. doi: 10.1016/j.stemcr.2019.06.005, 31353226 PMC6700675

[108] Saravia J Raynor JL Chapman NM Lim SA Chi H. Signaling networks in immunometabolism. Cell Res. (2020) 30:328–42. doi: 10.1038/s41422-020-0301-1, 32203134 PMC7118125

[109] Cho CH Patel S Rajbhandari P. Adipose tissue lipid metabolism: lipolysis. Curr Opin Genet Dev. (2023) 83:102114 37738733 10.1016/j.gde.2023.102114 PMC12834039

[110] Fabbrini E Sullivan S Klein S. Obesity and nonalcoholic fatty liver disease: biochemical, metabolic, and clinical implications. Hepatology. (2010) 51:679–89. doi: 10.1002/hep.23280, 20041406 PMC3575093

[111] Thomas D Apovian C. Macrophage functions in lean and obese adipose tissue. Metabolism. (2017) 72:120–43. doi: 10.1016/j.metabol.2017.04.005, 28641779 PMC5516622

[112] Anton SD Moehl K Donahoo WT Marosi K Lee SA Mainous AG 3rd . Flipping the metabolic switch: understanding and applying the health benefits of fasting. Obesity. (2018) 26:254–68. doi: 10.1002/oby.22065, 29086496 PMC5783752

[113] Duregon E Pomatto-Watson LCDD Bernier M Price NL de Cabo R. Intermittent fasting: from calories to time restriction. Geroscience. (2021) 43:1083–92. doi: 10.1007/s11357-021-00335-z, 33686571 PMC8190218

[114] Wang X Shen X Yan Y Li H. Pyruvate dehydrogenase kinases (PDKs): an overview toward clinical applications. Biosci Rep. (2021) 41:4402. doi: 10.1042/BSR20204402, 33739396 PMC8026821

[115] Bertholdt L Gudiksen A Stankiewicz T Villesen I Tybirk J van Hall G . Impact of training state on fasting-induced regulation of adipose tissue metabolism in humans. J Appl Physiol. (2018) 124:729–40. doi: 10.1152/japplphysiol.00664.2017, 29191981

[116] Spriet LL Tunstall RJ Watt MJ Mehan KA Hargreaves M Cameron-Smith D. Pyruvate dehydrogenase activation and kinase expression in human skeletal muscle during fasting. J Appl Physiol. (2004) 96:2082–7. 14966024 10.1152/japplphysiol.01318.2003

[117] Shimazu T Hirschey MD Newman J He W Shirakawa K le Moan N . Suppression of oxidative stress by β-hydroxybutyrate, an endogenous histone deacetylase inhibitor. Science. (2013) 339:211–4. doi: 10.1126/science.1227166, 23223453 PMC3735349

[118] Carretta MD . Hydroxycarboxylic acid receptor 2 (HCA2) agonists induce NET formation and MMP-9 release from bovine polymorphonuclear leukocytes. Develop Comp Immunol. (2023) 139:104562 10.1016/j.dci.2022.104562 36183839

[119] Li C Zhang H Wu H Li R Wen D Tang Y . Intermittent fasting reverses the declining quality of aged oocytes. Free Radic Biol Med. (2023) 195:74–88. doi: 10.1016/j.freeradbiomed.2022.12.084, 36581058

[120] Navas LE Carnero A. NAD+ metabolism, stemness, the immune response, and cancer. Signal Transduct Target Ther. (2021) 6:2. doi: 10.1038/s41392-020-00354-w, 33384409 PMC7775471

[121] Zhao T Fan J Abu-Zaid A Burley S Zheng XF. Nuclear mTOR signaling orchestrates transcriptional programs underlying cellular growth and metabolism. Cells. (2024) 13:781. doi: 10.3390/cells13090781, 38727317 PMC11083943

[122] Chi H. Regulation and function of mTOR signalling in T cell fate decisions. Nat Rev Immunol. (2012) 12:325–38. doi: 10.1038/nri3198, 22517423 PMC3417069

[123] Araki K Turner AP Shaffer VO Gangappa S Keller SA Bachmann MF . mTOR regulates memory CD8 T-cell differentiation. Nature. (2009) 460:108–12. doi: 10.1038/nature08155, 19543266 PMC2710807

[124] Fan J Khanzada Z Xu Y. Mechanisms underlying muscle-related diseases and aging: insights into pathophysiology and therapeutic strategies. Muscles. (2025) 4:26. doi: 10.3390/muscles4030026, 40843913 PMC12371960

[125] Fan J Yuan Z Burley SK Libutti SK Zheng XFS. Amino acids control blood glucose levels through mTOR signaling. Eur J Cell Biol. (2022) 101:151240. doi: 10.1016/j.ejcb.2022.151240, 35623230 PMC10035058

[126] Brandhorst S Choi IY Wei M Cheng CW Sedrakyan S Navarrete G . A periodic diet that mimics fasting promotes multi-system regeneration, enhanced cognitive performance, and healthspan. Cell Metab. (2015) 22:86–99. doi: 10.1016/j.cmet.2015.05.012, 26094889 PMC4509734

[127] Sarafidou K Alexakou E Talioti E Bakopoulou A Anastassiadou V. The oral microbiome in older adults–a state-of-the-art review. Archives Gerontology Geriatrics Plus. (2024) 1:100061. doi: 10.1016/j.aggp.2024.100061

[128] Kazarina A Kuzmicka J Bortkevica S Zayakin P Kimsis J Igumnova V . Oral microbiome variations related to ageing: possible implications beyond oral health. Arch Microbiol. (2023) 205:116. doi: 10.1007/s00203-023-03464-5, 36920536 PMC10016173

[129] Peng X Cheng L You Y Tang C Ren B Li Y . Oral microbiota in human systematic diseases. Int J Oral Sci. (2022) 14:14. doi: 10.1038/s41368-022-00163-7, 35236828 PMC8891310

[130] Azzolino D Felicetti A Santacroce L Lucchi T Garcia-Godoy F Passarelli PC. The emerging role of oral microbiota: a key driver of oral and systemic health. Am J Dent. (2025) 38:111–6. 40455948

[131] Turnbaugh PJ Ley RE Hamady M Fraser-Liggett CM Knight R Gordon JI. The human microbiome project. Nature. (2007) 449:804–10. 17943116 10.1038/nature06244 PMC3709439

[132] Jia G Zhi A Lai PFH Wang G Xia Y Xiong Z . The oral microbiota–a mechanistic role for systemic diseases. Br Dent J. (2018) 224:447–55. doi: 10.1038/sj.bdj.2018.217, 29569607

[133] Casati M . Gut microbiota and physical frailty through the mediation of sarcopenia. Exp Gerontol. (2019) 124:110639 31226349 10.1016/j.exger.2019.110639

[134] Azzolino D Carnevale-Schianca M Bottalico L Colella M Felicetti A Perna S . The Oral–gut microbiota Axis as a mediator of frailty and sarcopenia. Nutrients. (2025) 17:2408. doi: 10.3390/nu17152408, 40805993 PMC12348868

[135] Tan X Wang Y Gong T. The interplay between oral microbiota, gut microbiota and systematic diseases. J Oral Microbiol. (2023) 15:2213112. doi: 10.1080/20002297.2023.2213112, 37200866 PMC10187086

[136] Park S-Y Hwang BO Lim M Ok SH Lee SK Chun KS . Oral–gut microbiome axis in gastrointestinal disease and cancer. Cancer. (2021) 13:2124. doi: 10.3390/cancers13092124, 33924899 PMC8125773

[137] Costa CF . The oral-gut microbiota relationship in healthy humans: identifying shared bacteria between environments and age groups. Front Microbiol. (2024) 15:1475159. 39512939 10.3389/fmicb.2024.1475159 PMC11540997

[138] Kunath BJ de Rudder C Laczny CC Letellier E Wilmes P. The oral–gut microbiome axis in health and disease. Nat Rev Microbiol. (2024) 22:791–805. doi: 10.1038/s41579-024-01075-5, 39039286

[139] Imhann F Bonder MJ Vich Vila A Fu J Mujagic Z Vork L . Proton pump inhibitors affect the gut microbiome. Gut. (2016) 65:740–8. doi: 10.1136/gutjnl-2015-310376, 26657899 PMC4853569

[140] Xu Q Wang W Li Y Cui J Zhu M Liu Y . The oral-gut microbiota axis: a link in cardiometabolic diseases. NPJ Biofilms Microbiomes. (2025) 11:11. doi: 10.1038/s41522-025-00646-5, 39794340 PMC11723975

[141] Mukherjee S Chopra A Karmakar S Bhat SG. Periodontitis increases the risk of gastrointestinal dysfunction: an update on the plausible pathogenic molecular mechanisms. Crit Rev Microbiol. (2025) 51:187–217. doi: 10.1080/1040841X.2024.2339260, 38602474

[142] Shaffer M Lozupone C. Prevalence and source of fecal and oral bacteria on infant, child, and adult hands. Msystems. (2018) 3. doi: 10.1128/msystems.00192-17 PMC5768791 29359197

[143] Arimatsu K Yamada H Miyazawa H Minagawa T Nakajima M Ryder MI . Oral pathobiont induces systemic inflammation and metabolic changes associated with alteration of gut microbiota. Sci Rep. (2014) 4:4828. doi: 10.1038/srep04828, 24797416 PMC4010932

[144] Xiao L . Experimental periodontitis deteriorated atherosclerosis associated with trimethylamine N-oxide metabolism in mice. Front Cell Infect Microbiol. (2022) 11:820535 35118014 10.3389/fcimb.2021.820535 PMC8804528

[145] Guo X Shao Y. Role of the oral-gut microbiota axis in pancreatic cancer: a new perspective on tumor pathophysiology, diagnosis, and treatment. Mol Med. (2025) 31:103. doi: 10.1186/s10020-025-01166-w, 40102723 PMC11917121

[146] Leonov GE Varaeva YR Livantsova EN Starodubova AV. The complicated relationship of short-chain fatty acids and oral microbiome: a narrative review. Biomedicine. (2023) 11:2749. doi: 10.3390/biomedicines11102749, 37893122 PMC10604844

[147] Wu J-T . Oral short-chain fatty acids administration regulates innate anxiety in adult microbiome-depleted mice. Neuropharmacology. (2022) 214:109140 35613660 10.1016/j.neuropharm.2022.109140

[148] Guan X Li W Meng H. A double-edged sword: role of butyrate in the oral cavity and the gut. Mol Oral Microbiol. (2021) 36:121–31. doi: 10.1111/omi.12322, 33155411

[149] Asai S Nakamura Y Yamamura M Ikezawa H Namikawa I. Quantitative analysis of the Epstein-Barr virus-inducing properties of short-chain fatty acids present in the culture fluids of oral bacteria. Arch Virol. (1991) 119:291–6. doi: 10.1007/BF01310678, 1652240

[150] Adil NA Omo-Erigbe C Yadav H Jain S. The oral–gut microbiome–brain axis in cognition. Microorganisms. (2025) 13:814. doi: 10.3390/microorganisms13040814, 40284650 PMC12029813

[151] Wong JM de Souza R Kendall CW Emam A Jenkins DJ. Colonic health: fermentation and short chain fatty acids. J Clin Gastroenterol. (2006) 40:235–43. doi: 10.1097/00004836-200603000-00015, 16633129

[152] den Besten G Lange K Havinga R van Dijk TH Gerding A van Eunen K . *Gut-derived short-chain fatty acids are vividly assimilated into host carbohydrates and lipids.* American journal of physiology-gastrointestinal and liver. Physiology. (2013) 305:G900–10. doi: 10.1152/ajpgi.00265.2013 24136789

[153] Den Besten G . The role of short-chain fatty acids in the interplay between diet, gut microbiota, and host energy metabolism. J Lipid Res. (2013) 54:2325–40. 23821742 10.1194/jlr.R036012 PMC3735932

[154] Sonnenburg JL Bäckhed F. Diet–microbiota interactions as moderators of human metabolism. Nature. (2016) 535:56–64. doi: 10.1038/nature18846, 27383980 PMC5991619

[155] Ogawa T Hirose Y Honda-Ogawa M Sugimoto M Sasaki S Kibi M . Composition of salivary microbiota in elderly subjects. Sci Rep. (2018) 8:414. doi: 10.1038/s41598-017-18677-0, 29323208 PMC5765146

[156] Wells PM Sprockett DD Bowyer RCE Kurushima Y Relman DA Williams FMK . Influential factors of saliva microbiota composition. Sci Rep. (2022) 12:18894. doi: 10.1038/s41598-022-23266-x, 36344584 PMC9640688

[157] Thaiss CA Zeevi D Levy M Zilberman-Schapira G Suez J Tengeler AC . Transkingdom control of microbiota diurnal oscillations promotes metabolic homeostasis. Cell. (2014) 159:514–29. doi: 10.1016/j.cell.2014.09.048, 25417104

[158] Kaczmarek JL Musaad SM Holscher HD. Time of day and eating behaviors are associated with the composition and function of the human gastrointestinal microbiota. Am J Clin Nutr. (2017) 106:1220–31. doi: 10.3945/ajcn.117.156380, 28971851

[159] Zarrinpar A Chaix A Yooseph S Panda S. Diet and feeding pattern affect the diurnal dynamics of the gut microbiome. Cell Metab. (2014) 20:1006–17. doi: 10.1016/j.cmet.2014.11.008, 25470548 PMC4255146

[160] Gao Y Zhang H Fang K Yao Y Chen J Lu H . The relationship between frailty, BMI, and mortality in older adults: results from the CLHLS. BMC Geriatr. (2025) 25:539. doi: 10.1186/s12877-025-06197-w, 40684122 PMC12275446

[161] Li B Li Y Zhang Y Liu P Song Y Zhou Y . Visceral fat obesity correlates with frailty in middle-aged and older adults. Diabetes Metabolic Syndrome Obesity. (2022) 15:2877–84. doi: 10.2147/DMSO.S383597, 36164455 PMC9508679

[162] Yuan L Chang M Wang J. Abdominal obesity, body mass index and the risk of frailty in community-dwelling older adults: a systematic review and meta-analysis. Age Ageing. (2021) 50:1118–28. doi: 10.1093/ageing/afab039, 33693472

[163] Li CW Yu K Shyh-Chang N Jiang Z Liu T Ma S . Pathogenesis of sarcopenia and the relationship with fat mass: descriptive review. J Cachexia Sarcopenia Muscle. (2022) 13:781–94. doi: 10.1002/jcsm.12901, 35106971 PMC8977978

[164] Silay K Selvi Oztorun H. Sarcopenic obesity is linked to worse clinical outcomes than sarcopenia or obesity alone in hospitalized older adults. BMC Geriatr. (2025) 25:443. doi: 10.1186/s12877-025-06105-2, 40604522 PMC12219870

[165] Wei S . Sarcopenic obesity: epidemiology, pathophysiology, cardiovascular disease, mortality, and management. Front Endocrinol. (2023) 14:1185221. 10.3389/fendo.2023.1185221 PMC10344359 37455897

[166] Uchida K Sugimoto T Tange C Nishita Y Shimokata H Saji N . Association between abdominal adiposity and cognitive decline in older adults: a 10-year community-based study. J. Nutrition Health Aging. (2024) 28:100175. doi: 10.1016/j.jnha.2024.100175, 38308924 PMC12880568

[167] Yang M Hu M Zhang Y Jia S Sun X Zhao W . Sarcopenic obesity is associated with frailty among community-dwelling older adults: findings from the WCHAT study. BMC Geriatr. (2022) 22:863. doi: 10.1186/s12877-022-03617-z, 36384475 PMC9667677

[168] Yin D Li Y Liao X Tian D Xu Y Zhou C . FTO: a critical role in obesity and obesity-related diseases. Br J Nutr. (2023) 130:1657–64. doi: 10.1017/S0007114523000764, 36944362

[169] Czajkowski P Adamska-Patruno E Bauer W Fiedorczuk J Krasowska U Moroz M . The impact of FTO genetic variants on obesity and its metabolic consequences is dependent on daily macronutrient intake. Nutrients. (2020) 12:3255. doi: 10.3390/nu12113255, 33114268 PMC7690875

[170] Madkour MI . Ramadan diurnal intermittent fasting is associated with attenuated FTO gene expression in subjects with overweight and obesity: a prospective cohort study. Front Nutr. (2022) 8:741811 35372458 10.3389/fnut.2021.741811 PMC8968860

[171] Jahrami HA Faris ME I Janahi A I Janahi M Abdelrahim DN Madkour MI . Does four-week consecutive, dawn-to-sunset intermittent fasting during Ramadan affect cardiometabolic risk factors in healthy adults? A systematic review, meta-analysis, and meta-regression. Nutr Metab Cardiovasc Dis. (2021) 31:2273–301. doi: 10.1016/j.numecd.2021.05.002, 34167865

[172] Faris MAIE . Impact of Ramadan intermittent fasting on oxidative stress measured by urinary 15-F2t-Isoprostane. J Nutrition Metabolism. (2012) 2012:802924. doi: 10.1155/2012/802924, 23150812 PMC3485525

[173] Annapoorna P Iyer H Parnaik T Narasimhan H Bhattacharya A Kumar A. FTO: an emerging molecular player in neuropsychiatric diseases. Neuroscience. (2019) 418:15–24. doi: 10.1016/j.neuroscience.2019.08.021, 31442565

[174] Lan N . FTO–a common genetic basis for obesity and cancer. Front Genet. (2020) 11:559138 33304380 10.3389/fgene.2020.559138 PMC7701174

[175] Woo A Botta A Shi SSW Paus T Pausova Z. Obesity-related neuroinflammation: magnetic resonance and microscopy imaging of the brain. Int J Mol Sci. (2022) 23:8790. doi: 10.3390/ijms23158790, 35955925 PMC9368789

[176] Lorena FB do Nascimento BPP Camargo ELRA Bernardi MM Fukushima AR do N Panizza J . Long-term obesity is associated with depression and neuroinflammation. Archives Endocrinol Metabolism. (2021) 65:537–48. doi: 10.20945/2359-3997000000400, 34714995 PMC10528574

[177] Lin R Liu W Piao M Zhu H. A review of the relationship between the gut microbiota and amino acid metabolism. Amino Acids. (2017) 49:2083–90. doi: 10.1007/s00726-017-2493-3, 28932911

[178] Li M Yang Y Chen T Luo Y Zhang Y Liu H . FTO (fat-mass and obesity-associated protein) deficiency aggravates age-dependent depression-like behaviors and cognitive impairment. Behav Brain Funct. (2025) 21:18. doi: 10.1186/s12993-025-00280-3, 40518522 PMC12167586

[179] Hayley S Hakim AM Albert PR. Depression, dementia and immune dysregulation. Brain. (2021) 144:746–60. doi: 10.1093/brain/awaa405, 33279966 PMC8041341

[180] Seidler K Barrow M. Intermittent fasting and cognitive performance–targeting BDNF as potential strategy to optimise brain health. Front Neuroendocrinol. (2022) 65:100971 34929259 10.1016/j.yfrne.2021.100971

[181] Couto S Cenit MC Montero J Iguacel I. The impact of intermittent fasting and Mediterranean diet on older adults physical health and quality of life: a randomized clinical trial. Nutr Metab Cardiovasc Dis. (2025) 35:104132. doi: 10.1016/j.numecd.2025.104132, 40451678

[182] Vernieri C Fucà G Ligorio F Huber V Vingiani A Iannelli F . Fasting-mimicking diet is safe and reshapes metabolism and antitumor immunity in patients with cancer. Cancer Discov. (2022) 12:90–107. doi: 10.1158/2159-8290.CD-21-0030, 34789537 PMC9762338

[183] Qian J Fang Y Yuan N Gao X Lv Y Zhao C . Innate immune remodeling by short-term intensive fasting. Aging Cell. (2021) 20:e13507. doi: 10.1111/acel.13507, 34705313 PMC8590100

[184] Chen Y . Time-restricted eating reveals a “younger” immune system and reshapes the intestinal microbiome in human. Redox Biol. (2024) 78:103422 39561680 10.1016/j.redox.2024.103422 PMC11616606

[185] Longo VD Panda S. Fasting, circadian rhythms, and time-restricted feeding in healthy lifespan. Cell Metab. (2016) 23:1048–59. doi: 10.1016/j.cmet.2016.06.001, 27304506 PMC5388543

[186] Ferrucci L Fabbri E. Inflammageing: chronic inflammation in ageing, cardiovascular disease, and frailty. Nat Rev Cardiol. (2018) 15:505–22. doi: 10.1038/s41569-018-0064-2, 30065258 PMC6146930

[187] Anton S . The effects of intermittent fasting regimens in middle-age and older adults: current state of evidence. Exp Gerontol. (2021) 156:111617 34728336 10.1016/j.exger.2021.111617

[188] Zhong F Zhu T Jin X Chen X Wu R Shao L . Adverse events profile associated with intermittent fasting in adults with overweight or obesity: a systematic review and meta-analysis of randomized controlled trials. Nutr J. (2024) 23:72. doi: 10.1186/s12937-024-00975-9, 38987755 PMC11234547

[189] Currenti W Buscemi S Cincione RI Cernigliaro A Godos J Grosso G . Time-restricted feeding and metabolic outcomes in a cohort of Italian adults. Nutrients. (2021) 13:1651. doi: 10.3390/nu13051651, 34068302 PMC8153259

[190] Trepanowski JF Kroeger CM Barnosky A Klempel MC Bhutani S Hoddy KK . Effect of alternate-day fasting on weight loss, weight maintenance, and cardioprotection among metabolically healthy obese adults: a randomized clinical trial. JAMA Intern Med. (2017) 177:930–8. doi: 10.1001/jamainternmed.2017.0936, 28459931 PMC5680777

[191] Wu C Chen B Yu J Zhang Q Piao C. Effect of the 5: 2 diet on weight loss and cardiovascular disease risk factors in overweight and/or obesity: a systematic review and Meta-analysis. Int J Endocrinol. (2025) 2025:6658512. doi: 10.1155/ije/6658512, 40041761 PMC11876533

[192] Liu Z-L Chen HH Zheng LL Sun LP Shi L. Angiogenic signaling pathways and anti-angiogenic therapy for cancer. Signal Transduct Target Ther. (2023) 8:198. doi: 10.1038/s41392-023-01460-1, 37169756 PMC10175505

[193] Ezzati A Tamargo JA Golberg L Haub MD Anton SD. The effects of time-restricted eating on inflammation and oxidative stress in overweight older adults: a pilot study. Nutrients. (2025) 17:322. doi: 10.3390/nu17020322, 39861451 PMC11768921

