# Correction: Intermittent fasting and immune aging: implications for immunosenescence, inflammaging, neuroinflammation, and frailty

**DOI:** 10.3389/fnut.2026.1797601

**Published:** 2026-02-09

**Authors:** Dania Alkawamleh, Mohamed I. Madkour, Faiza Kalam, Dana N. Abdelrahim, Hanan Wael Abdallah, MoezAlIslam E. Faris

**Affiliations:** 1 Department of Nutrition, King Hussein Cancer Center, Amman, Jordan; 2 Department of Clinical Nutrition and Dietetics, Faculty of Allied Medical Sciences, Applied Science Private University, Amman, Jordan; 3 Department of Medical Laboratory Sciences, College of Health Sciences, University of Sharjah, Sharjah, United Arab Emirates; 4 Research Institute of Medical and Health Sciences (RIMHS), University of Sharjah, Sharjah, United Arab Emirates; 5 Division of Cancer Prevention and Control, Department of Internal Medicine, The Ohio State University, Columbus, OH, United States; 6 Ohio State University Comprehensive Cancer Center, The Ohio State University, Columbus, OH, United States

**Keywords:** AMPK activation, healthy aging, immune resilience, inflammasome, mTOR signaling, time-restricted eating

The funder, The Applied Science Private University, Amman, Jordan, Grant Number: DRGS-DRGS/2025/11 to MoezAlIslam E. Faris was erroneously omitted. The correct Funding statement appears below.

The original version of this article has been updated.

